# The Effect of Water on the 2‐Propanol Oxidation Activity of Co‐Substituted LaFe_1−x_Co_x_O_3_ Perovskites

**DOI:** 10.1002/chem.202102791

**Published:** 2021-11-08

**Authors:** Maik Dreyer, Daniel Cruz, Ulrich Hagemann, Patrick Zeller, Markus Heidelmann, Soma Salamon, Joachim Landers, Anna Rabe, Klaus Friedel Ortega, Sharif Najafishirtari, Heiko Wende, Nils Hartmann, Axel Knop‐Gericke, Robert Schlögl, Malte Behrens

**Affiliations:** ^1^ Faculty for Chemistry and Center for Nanointegration Duisburg-Essen (CENIDE) University of Duisburg-Essen Universitätsstr. 7 45141 Essen Germany; ^2^ Department of Inorganic Chemistry Fritz-Haber-Institut der Max-Planck Gesellschaft Faradayweg 4–6 14195 Berlin Germany; ^3^ Department of Heterogeneous Reactions Max Planck Institute for Chemical Energy Conversion Stiftstraße 34–36 Mülheim an der Ruhr 45470 Germany; ^4^ Interdisciplinary Center for Analytics on the Nanoscale (ICAN) NanoEnergieTechnikZentrum at University of Duisburg-Essen Carl-Benz-Str. 199 47057 Duisburg Germany; ^5^ Helmholtz-Zentrum Berlin für Materialien und Energie GmbH BESSY II Department of Catalysis for Energy Albert-Einstein-Straße 15 12489 Berlin Germany; ^6^ Faculty of Physics and CENIDE University of Duisburg-Essen Lotharstr. 1 47057 Duisburg Germany; ^7^ Institute of Inorganic Chemistry Christian-Albrechts-Universität zu Kiel Max-Eyth-Straße 2 24118 Kiel Germany

**Keywords:** 2-Propanol, LFCO, Oxidation, Oxygen Vacancies, Perovskite phases

## Abstract

Perovskites are interesting oxidation catalysts due to their chemical flexibility enabling the tuning of several properties. In this work, we synthesized LaFe_1−x_Co_x_O_3_ catalysts by co‐precipitation and thermal decomposition, characterized them thoroughly and studied their 2‐propanol oxidation activity under dry and wet conditions to bridge the knowledge gap between gas and liquid phase reactions. Transient tests showed a highly active, unstable low‐temperature (LT) reaction channel in conversion profiles and a stable, less‐active high‐temperature (HT) channel. Cobalt incorporation had a positive effect on the activity. The effect of water was negative on the LT channel, whereas the HT channel activity was boosted for x>0.15. The boost may originate from a slower deactivation rate of the Co^3+^ sites under wet conditions and a higher amount of hydroxide species on the surface comparing wet to dry feeds. Water addition resulted in a slower deactivation for Co‐rich catalysts and higher activity in the HT channel state.

## Graphical abstract

**The effect of water addition on 2‐propanol oxidation** was studied on LaFe_1‐x_Co_x_O_3_ catalysts. After surpassing a Co content threshold (x=0.15) the effect of water on the activity at elevated temperatures is beneficial due to slower Co^3+^ reduction and the number of hydroxide species on the catalyst surface increased.

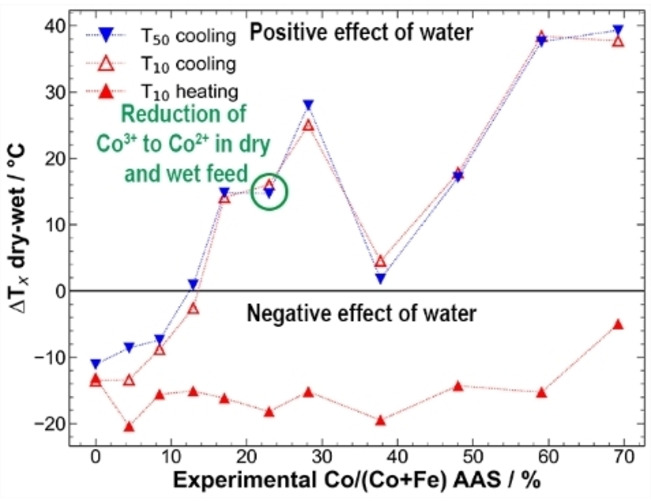

## Introduction

Oxidation catalysis is essential in the chemical industry, either to selectively synthesize products like oxygenates or to remove volatile organic compounds (VOCs) or carbon monoxide (CO) from exhaust gases.^[1]^ However, these reactions are often performed using noble metals as catalysts, which are very cost‐intensive due to their low abundance. Among potential oxide catalysts to perform those oxidation reactions at a more favorable cost are perovskite oxides with the general empirical formula ABO_3_.^[2]^ Due to their ability to incorporate different redox‐active transition metal cations on the B‐sites, these compounds are interesting materials to study the effect of composition on the catalytic properties. Additionally, they offer significant structural stability during oxidation catalysis.^[3]^ Furthermore, it is possible to tune the oxygen vacancies within their structures and thus the catalytic oxidation properties by manipulating the type and composition of the A‐cation, as reported for instance by replacing La^3+^ with Sr^2+^.^[4]^ As a probe reaction for selective oxidation and the removal of VOCs, the oxidation of 2‐propanol was widely studied on precious metals and oxides, as it features the possibility to gain mechanistic insights and to characterize Lewis acidic and Lewis basic surface sites.^[5]^

**Scheme 1:**
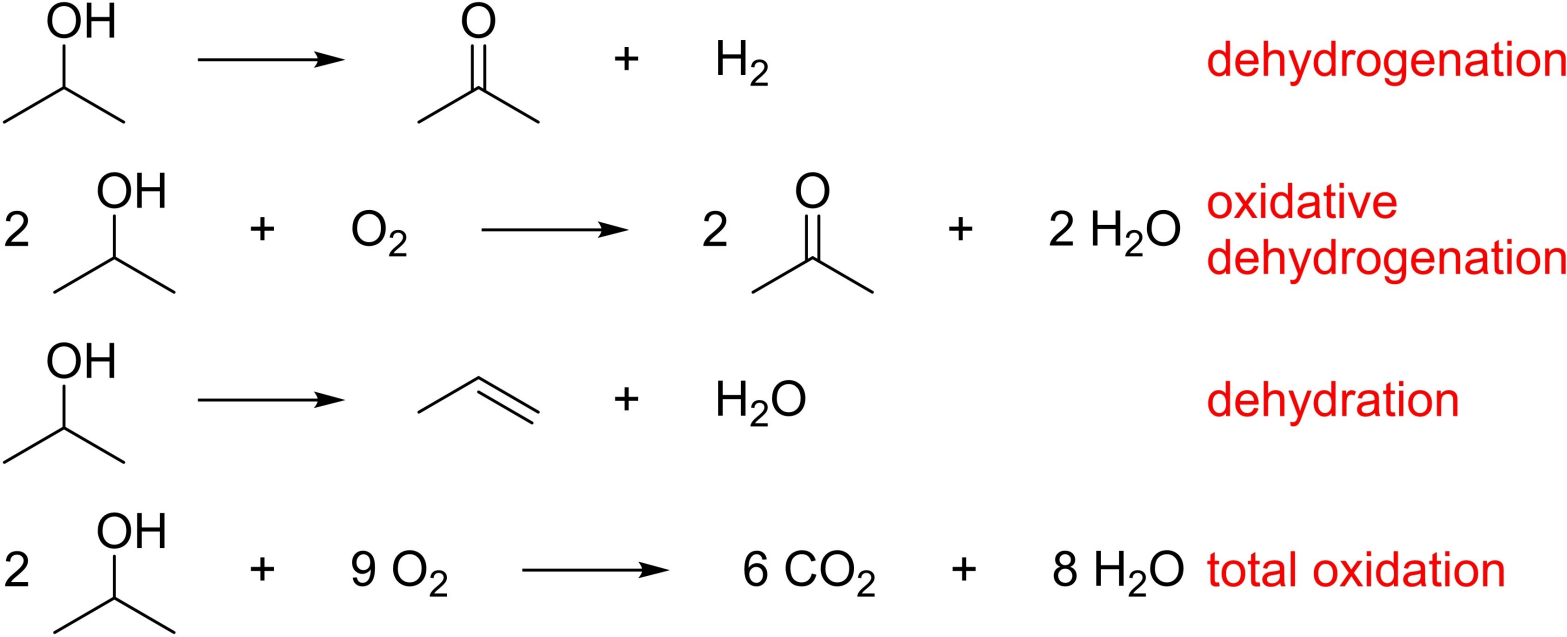
Possible reactions during the gas‐phase 2‐propanol oxidation.^[6]^

The main reactions taking place during the gas‐phase 2‐propanol oxidation are shown in Scheme 1. 2‐propanol reacts at strongly basic sites and moderately acidic sites in their vicinity to the desired selective oxidation product, i. e., acetone and hydrogen, via the dehydrogenation pathway. The oxidative dehydrogenation is catalyzed by the same centers but features oxidation to acetone and water as the coupled product. The dehydration is catalyzed by strongly acidic sites in the vicinity of weakly basic sites, leading to propene and water as reaction products. Another pathway is total oxidation, in which CO_2_ is formed at high temperatures.^[6]^

Based on the recent work by Anke et al.,^[5b]^ the mechanism of gas‐phase 2‐propanol oxidation on Co_3_O_4_ was described to take place on 5‐fold‐coordinated octahedral Co^3+^ on the surface as the active site for adsorption of both 2‐propanol and the reactive oxygen species. Oxidative dehydrogenation was found to be the favored pathway facilitated by the adsorption of atomic oxygen. Furthermore, the reduction of Co^3+^ to Co^2+^ was observed during the reaction, which led to a deactivation of the catalyst. The reaction showed different pathways, a non‐stable low temperature (LT) pathway with high activity only during heating and a stable high‐temperature (HT) pathway with lower activity. For spinels, a high amount of exposed (110) surface seems to be beneficial for high activity and stability.^[7]^

By sum‐frequency spectroscopy, the adsorption of 2‐propanol on TiO_2_ was detected to take place molecularly.^[8]^ On a SrTiO_3_ perovskite with a mainly exposed (100) facet, an orientation of the alcohol with the C−H group pointing to the surface was found. The resulting geometry enabled the abstraction of an α‐proton during the dehydrogenation pathway.^[5f]^ Reaction selectivity of SrTiO_3_ was dependent on the pretreatment, caused by surface restructuring due to segregation.[^5d^, ^9^]

The effect of water was previously studied in the 2‐propanol oxidation reaction on spinels and noble metals. Water adsorption occurs on strongly acidic sites and is expected to suppress dehydration and improve dehydrogenation selectivity for oxide catalysts in oxidation reactions.^[6]^ Therefore, water addition should lead to a more pronounced (oxidative) dehydrogenation mechanism due to competitive adsorption on strongly acidic sites. Additionally, by using a wet feed with a high concentration of water, one could take one step further from gas‐phase catalysis towards the liquid‐phase and therefore facilitate understanding of the liquid‐phase oxidation. Similarities between 2‐propanol oxidation on Co_3_O_4_ in the liquid phase and the gas phase with water vapor were reported.^[10]^ Also, a detrimental effect of water feeding on the low‐temperature activity of gas‐phase 2‐propanol oxidation was found in the same study.^[10]^ On Pt catalysts, water in the gas‐mixture was studied for different alcohol oxidations. The addition of water resulted in decreasing conversions and selective oxidation selectivity for the oxidation of ethanol.^[11]^ For 1‐propanol oxidation, a steady decay in conversion was observed, but no stable trend in the ratio of total and selective oxidation products. In addition, an increase in activation energy was reported for water co‐feeding.^[12]^ For the oxidation of 2‐propanol on Pt nanoparticles, a steady decay in conversion with increasing amount of water in the feed was observed.^[13]^

For perovskites, the influence of water on different oxidation reactions was reported and mostly had the effect of reduced conversion at the same temperature, as will be discussed in more detail in the following paragraph. In CO and toluene oxidation on La_0.6_Sr_0.4_MnO_3_ perovskites, water decreased the conversion.^[14]^ For methane oxidation on La_1−x_Sr_x_FeO_3_ catalysts, a decrease in conversion with water concentration was found for lower temperatures, whereas no deactivation was observed at elevated temperatures.^[15]^ In ethanol oxidation over an octahedral molecular sieve (OMS‐2) catalyst, the addition of 10 % water led to a pronounced decrease in activity and was attributed to competitive adsorption of water and ethanol on the active sites.^[16]^ A similar conclusion was obtained from *n*‐butane oxidation over vanadium phosphorous oxide. However, in this specific case, competitive adsorption of water and oxygen led to a difference in oxygen activation.^[17]^ In the parallel oxidation of NO and propane, a loss in activity with water addition was reported and correlated to competitive adsorption of oxygen, NO and water.^[18]^ Also, the filling of oxygen vacancies by water was considered a possible explanation for activity reduction. Interestingly, the extent of deactivation of both reactions was less severe for LaCoO_3_ than for LaFeO_3_ and attributed to more abundant anion vacancies in the Co‐containing perovskite_,_ and the activity decrease was depressed with rising temperature.^[18]^ In terms of VOC removal, the effect of water is complex, especially at low temperatures, and depends on many factors.^[1c]^

Perovskite oxides can be synthesized in various compositions and by various methods.^[2]^ Recently, we presented the synthesis of LaFe_1−x_Co_x_O_3_ (LFCO) perovskites by co‐precipitation of a mostly amorphous precursor system and subsequent thermal decomposition.^[19]^ LCFO materials are interesting oxidation catalysts since the two transition metals Co and Fe are earth‐abundant and showed high catalytic activity in various oxidation reactions.^[20]^ Further exemplary methods for perovskite synthesis are spray‐flame synthesis and ceramic methods.[^3^, ^21^] In these materials, the A‐cation is the larger of the two cations, typically a halide or lanthanide element and is considered a weak acidic center in perovskite oxides. The octahedrally coordinated B‐cation is often a redox‐active metal and is the strong Lewis acidic center. The oxygen anion acts as a Lewis basic center.^[22]^

LSCF (lanthanum strontium cobalt iron) perovskites present acidic and basic surface sites. Upon Fe/Co substitution, non‐linear behavior of the surface basicity based on Co content was reported, but in general, a decreased basicity with iron content was found.^[23]^ For LaCoO_3_, an acidic surface was reported based on CO adsorption.[^2^, ^24^] La_2_O_3_ excess in mixed LaFe oxides leads to an increase in surface basicity in LaFeO_3_ perovskites shown by 2‐propanol decomposition, indicating an acidic surface of LaFeO_3_ and basic surface of La_2_O_3_.^[25]^

In terms of oxidation activity, especially Sr‐terminated step sites were found as very active sites in C−H bond activation of methane and CO oxidation which indicates an influence of the A‐cations on catalysis.^[26]^ Also, the effect of oxygen vacancies created upon Sr‐incorporation cannot be neglected.^[26c]^

In order to combine the reported effects of Co^3+^ sites on perovskite surfaces, we extended the previously reported synthesis protocol to LFCO materials with a nominal Co content of up to 70 % on the B‐site in this work.^[19]^ Adsorption of 2‐propanol was studied on selected materials, and gas‐phase 2‐propanol oxidation was performed with and without the co‐feeding of water vapor to study the effect of water on the catalytic properties as a function of composition. In this regard, two equally important goals were considered in this study, namely the effect of Co content on one hand and the effect of water in the feed on the other hand. In addition, the spent catalysts were characterized by X‐ray photoelectron spectroscopy (XPS) and scanning transmission electron microscopy (STEM) and compared to the initial state to gain insight into the dynamic changes of the catalysts during the reaction.

## Results and discussion

### Synthesis and materials characterization

Precursor materials were prepared via constant pH co‐precipitation in a semi‐automatized laboratory reactor, following the recipe reported in previous publications for a nominal Co content determined by x=Co/(Co+Fe) of 0.00≤x≤0.30.[^19^, ^27^] In this study, the Co‐content was extended up to x=0.70. The computer‐controlled precipitations were performed by simultaneous dosing of metal salt and base solutions, which allowed maintaining a constant pH during co‐precipitation. All synthesis protocols are shown in Figure S1.

The freshly prepared precipitated materials for x=0.00 and x=0.05 showed an X‐ray amorphous powder pattern, as shown in Figure S2a. For x>0.10, the formation of a hydrotalcite‐like secondary phase was additionally observed. The presence of the phase was indicated by the evolution of the most prominent (003) reflection of a CoFe‐layered double hydroxide (MgAl‐LDH structure type, ICSD reference code 6296^[28]^). The intensity of this reflection increased with increasing Co content in the precipitated metal salt solution. The experimental Co/(Co+Fe) ratio derived from atomic absorption spectroscopy of the precursor revealed a slight deficiency of Co compared to Fe in the materials after precipitation, as shown in Figure S2b.

**Figure 1:**
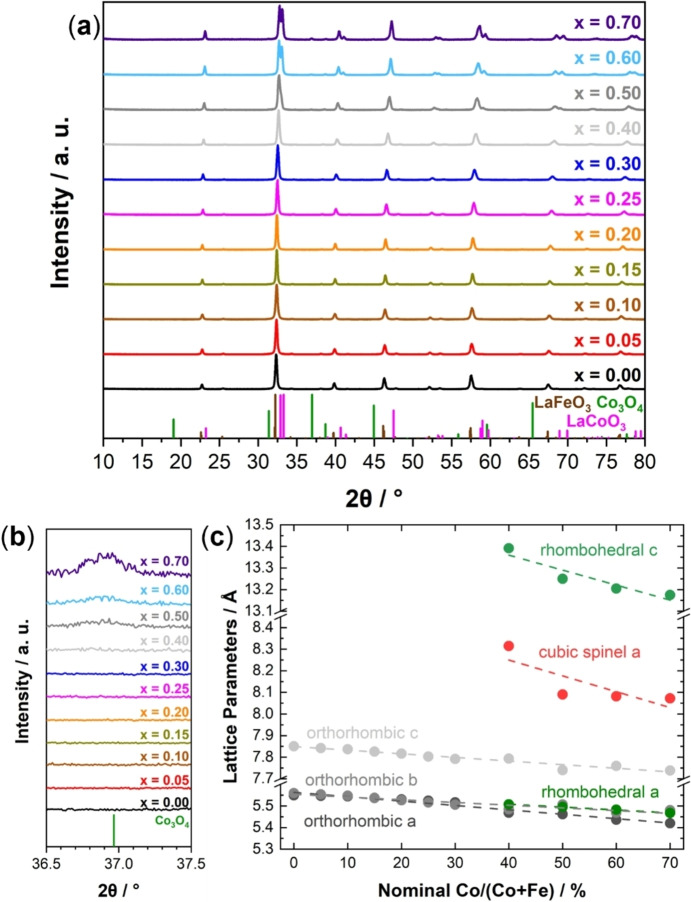
(a) XRD patterns of the materials after calcination. (b) Zoom into the XRD patterns after calcination, indicating a secondary spinel phase for x>0.30. (c) Evolution of the lattice parameters from Rietveld refinements.

Precursor materials were thermally decomposed by calcination at 800 °C and characterized via XRD with the resulting patterns shown in Figure 1a. The formerly amorphous precursors were transformed into crystalline materials. For x<0.40, only the orthorhombic perovskite phase was observed, indicated with a LaFeO_3_ reference (ICSD collection code 93611^[29]^). As reported in our previous study, the (112) reflection shifted to higher angles, indicating the incorporation of the smaller Co^3+^ cations into the perovskite lattice.[^19^, ^30^]

For x≥0.40, no phase pure materials were synthesized. Instead, a mixture of three crystalline phases was observed. An orthorhombic phase (like LaFeO_3_), a rhombohedral structure (like LaCoO_3_, ICSD collection code 99369^[31]^) and a spinel (indicated by Co_3_O_4_ ICSD collection code 9362^[32]^) were observed in the XRD patterns. The evolution of rhombohedral LaCoO_3_ is in good agreement with the literature, where the transition was also reported for x≥0.40 in LaFe_1−x_Co_x_O_3_.^[33]^ In addition, for x>0.40, a minor amount of Fe_3‐x_Co_x_O_4_ spinel phase was formed. A possible reason for this is cation segregation upon LDH formation. Therefore, there is no homogeneous cation distribution which leads to the spinel formation due to lack of La in the LDH.

The powder diffraction patterns were further analyzed by Rietveld refinement to determine lattice parameters of the different phases and the ratio of phases in the mixture.^[34]^ The refinement results are shown for each catalyst in Figure S3, the fit parameters in Table S1. The intermixing of the cationic sublattice was verified by refinement, as shown in Figure 1c. The lattice parameters for all three phases decreased linearly with increasing nominal Co fraction in the solid solution. Even though the XRD pattern of sample x=0.40 barely indicated the presence of the spinel phase, its existence was verified by the refinement. The peak position of the most intense reflection of the spinel phase corresponding to (311) planes in Figure 1b indicated the formation of Co‐richer spinels when more Co is precipitated.^[35]^ The lattice parameters of the spinel phase shown in Figure 1c indicate a Co‐rich spinel as well, but the spinel is not only consisting of Co, based on the literature lattice parameter of 8.065 Å for Co_3_O_4_.^[32]^ The lattice parameter of the spinels show the most prominent change between x=0.40 and x=0.50, but the general trend in the lattice parameter is also decreasing upon minor changes between x=0.50, x=0.60 and x=0.70. In Table 1, fractions of the phases are displayed. The fraction of orthorhombic perovskite decreases for x≥0.40, while the increasing amount of rhombohedral perovskite and spinel was confirmed by refinement.

**Table 1:** BET surface area determined by multi‐point BET plots, pore volumes determined at p/p_0_=0.99, pore sizes determined by the BJH method during desorption, and fractions of different phases derived from Rietveld refinements.

x=	BET surface area / m^2^ g^−1^	Pore volume / cm^3^ g^−1^	Fraction orthorhombic perovskite / %	Fraction rhombohedral perovskite / %	Fraction spinel / %
0.00	20.0	0.167	100	–	–
0.05	17.3	0.174	100	–	–
0.10	28.9	0.238	100	–	–
0.15	11.7	0.127	100	–	–
0.20	11.0	0.057	100	–	–
0.25	14.4	0.089	100	–	–
0.30	10.6	0.064	100	–	–
0.40	10.1	0.034	74	25	1
0.50	9.5	0.029	60	38	2
0.60	7.0	0.029	36	62	2
0.70	9.5	0.034	34	62	5

N_2_ physisorption isotherms and pore size distribution plots determined by the Barrett‐Joyner‐Halenda (BJH) method during desorption are shown in Figure S4 and Figure S5. All isotherms were characterized as type IVa isotherms.^[36]^ A decrease of the adsorbed volume was evident with an increasing amount of Co in the catalysts, even though there was a maximum in the adsorbed volume for x=0.10. The pore size distributions showed a maximum in the range of 20–30 nm for the samples up to x=0.30, indicating mainly interparticle pores, similar to what was reported in our previous work for samples up to x=0.30, where we described round but sintered particles and a BET surface area correlating well with the particle size.^[19]^ For the non‐phase pure samples x>0.30, the uniformity of the pore size distribution was lost, and no clear maximum was observed. For higher Co contents, the pore volume remained unaffected by the amount of cobalt in the material and decreased in this regime compared to the phase pure materials. BET surface area determined by multi‐point BET plots and pore volumes determined at p/p_0_=0.99 are shown in Table 1 and confirm the trends discussed based on the isotherms.

To obtain complementary structural information, including the Fe‐content of individual phases, as well as on Fe valence states, Mössbauer spectroscopy and magnetometry measurements were performed. Mössbauer spectra recorded at 5 K shown in Figure S6a display the expected sextet structure for the Fe‐bearing perovskites. For low Co‐contents x, we observed narrow sextet spectra, proving the complete Fe‐bearing material to be in a magnetically ordered state, which was reported to be weakly ferromagnetic^[37]^ (WFM), as also indicated in spectra obtained in external magnetic fields of 5 T (Figure S6b). Upon rising x, the spectra exhibited a minor decrease in sextet splitting (magnetic hyperfine field), with asymmetric line broadening assigned to different local surroundings of the individual Fe atoms and an isomer shift of ca. 0.47 mm s^−1^ relative to α‐Fe at room temperature, characteristic of the Fe^3+^ state.^[38]^ No further additional spectral features were observable that deviate from the hyperfine field or isomer shift of the above discussed perovskite contribution, which would indicate for example the presence of other Fe‐bearing phases. This agrees with results from XRD, indicating that the minor parasitic spinel phase is poor in Fe, being close to the Co_3_O_4_ stoichiometry.

In addition to the minor WFM hysteresis, field‐dependent magnetometry measurements at 4.3 K and 300 K (Figure S7) displayed a miniscule contribution with low coercive field and a spontaneous magnetization ≤0.5 Am^2^ kg^−1^ for some of the sample compositions, latter being especially visible in the 300 K M(H) curves. These could be assigned to a minute fraction (<1 %) of parasitic phase of high magnetization, for example, Fe‐rich spinels. The temperature‐dependent magnetization, normalized for better qualitative comparability and shown in Figure S7c, displayed a distinct shift in the perovskite Néel temperature from >400 K (x≈0.00) across ca. 300 K (x≈0.50) down to lower temperatures (x>0.50) based on peak features in the zero‐field cooled magnetization and convergence point of the field cooled and zero field cooled branches. The trend in Néel temperatures was similar to previous literature reports[^37b^, ^39^] for varying Co‐content in LaFe_1−x_Co_x_O_3_, which further supported the finding that macroscopic stoichiometries are principally preserved in the perovskite phase, which is of primary interest here.

TEM micrographs of the catalysts are shown in Figure S8 and Figure S9. The micrographs revealed the crystallinity of the catalysts and indicated a rather broad particle size distribution. Furthermore, the particles exhibited faceted shapes. Since the bulk composition of the precursor determined by AAS (Figure S2b) suggested only a minor bulk deficiency of Co, ratios of Co/(Co+Fe) and (Co+Fe)/La were determined by STEM‐EDX and XPS to gain information on the local and the surface composition of the perovskite catalysts. The Co/(Co+Fe) ratio of the as‐prepared materials, i. e., the calcined material prior to any catalytic experiment, is shown in Figure S10a. The surface ratio of Co/(Co+Fe) derived from XPS suggested a Fe‐enrichment and Co‐deficiency on the surface. Compared to STEM‐EDX analysis, which probes the surface and the bulk of the particles, nearly no deviation was observed, while in comparison to the precursors′ AAS results, both methods indicated a lower Co/(Co+Fe) ratio. The differences might result from the sample amount used for the analysis or different systematic errors of the individual measurements. Still, the deviation from the nominal composition might still be within the error of the measurement.

Furthermore, XPS indicated an enrichment of the B‐cations on the surface, as displayed in Figure S10b. In general, there are more redox‐active B‐cations on the surface compared to presumably redox‐inert A‐cations, inferred by the decrease of the (Co+Fe)/La ratio with increasing nominal Co content in the materials. For the catalysts considered to be phase‐pure orthorhombic perovskite (0.00≤x≤0.30), the B‐cation excess on the surface decreased strongly with increasing Co content. The decrease was less pronounced for the catalysts that showed the rhombohedral perovskite and spinel phases (0.40≤x≤0.70). It should be mentioned that the x=0.00 catalyst from the same series was studied in a previous work by high‐resolution STEM‐EDX and found to be B‐terminated.^[27]^

The bulk can be considered La rich for the whole range of Co contents (0.00≤x≤0.70), as seen from the STEM‐EDX data. In literature, an A‐cation enrichment on the perovskite surface is reported based on XPS after hydrothermal synthesis of SrTiO_3_.^[9]^ Low energy ion scattering with increased surface sensitivity indicated domination of A‐cations on the surface and a B‐cation enriched region below the surface, for example, for La_0.6_Sr_0.4_Co_0.2_Fe_0.8_O_3_. For LaFe_1‐x_Co_x_O_3_ materials, La enrichment on the surface was reported,^[38b]^ as well as on LaFeO_3_.^[41]^ However, STEM‐EDX measurements indicated an A‐cation enrichment as well on the surface.^[3a]^ In contrast, B‐cation enrichment is reported for La_1−x_Sr_x_NiO_3_ after thermal decomposition of amorphous citrate materials.^[42]^ For the catalysts discussed in this study, a B‐cation termination was found and reported in another story based on EDX mappings.^[27]^ There might be an effect of the synthesis method and perovskite composition on which cations are preferentially exposed on the surface, thus the B‐cation enrichment on the surface might evolve from the amorphous precursor decomposition approach.^[42]^

In summary, detailed characterization showed phase segregation for x>0.30 in orthorhombic perovskite, rhombohedral perovskite, and a spinel phase, as seen by XRD and confirmed by Mössbauer spectroscopy and magnetometry measurements. At lower Co contents, only the orthorhombic perovskite was observed. The phase segregation must be thoroughly considered when discussing catalytic properties. In addition, the materials were slightly Co‐deficient on the B‐site of the catalysts whereas for x≤0.30 an enrichment of B‐cations on the surface was observed.

### Adsorption and desorption of 2‐propanol

To investigate the interaction of the catalysts with different Co contents with 2‐propanol as one of the main reactants, its adsorption at 20 °C was used as a probe on the selected catalysts, i. e., x=0.00, x=0.10, x=0.25, and x=0.70, by diffuse reflectance infrared Fourier transform spectroscopy (DRIFTS). The evolution of the surface species for each catalyst during 40 min of adsorption after an oxidative treatment is shown in Figure S11. The presented times were representative of the amount of change that occurred in the spectra. The most prominent change was observed in the initial seconds while after 5 min, mainly gradual changes were observed in the intensity of the evolved species. Due to different IR absorption coefficients of the samples, the spectra were normalized based on their maximum peak intensity at 40 min.

**Figure 2:**
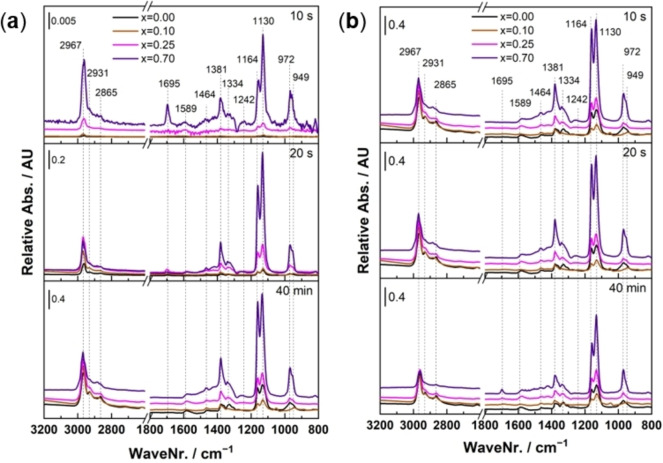
Comparison of DRIFTS spectra at selected times during (a) adsorption and (b) desorption of 2‐propanol.

The corresponding normalized spectra at 10 s, 20 s, and 40 min are compared in Figure 2a. The first two times were chosen to show the buildup of the species on the surface in the early state of the experiment, while the latest was chosen to show the maximum coverage of the adsorbed species on the surface. With increasing Co incorporation, the adsorption capacity increased, as was inferred from the intensities of the normalized spectra at initial times. Several bands were observed, which are attributed to different modes of bond vibrations, mainly because of the dissociative adsorption of 2‐propanol on the surface of the catalysts. However, the superimposition of the gas‐phase 2‐propanol bands, especially at longer times, could not be excluded.^[43]^

The bands at ∼2967 cm^−1^ and 2931 cm^−1^ are related to the asymmetric and symmetric stretching of ‐>CH_3_, respectively,^[44]^ while the band at 2865 cm^−1^ is attributed to the stretching of C−H bonds. Interestingly, by zooming in the spectra in this wavenumber range (Figure S11, insets), small but detectable variations among the samples were observed. Indeed, by increasing the Co content, two shoulders appeared and became more intense. This could mean that the electronic configuration of the adsorbed 2‐propanol varies among the samples because of their interactions with different adsorption sites. The observation is consistent with having multiple crystalline structures and phase impurities, especially at the highest Co content. Another band was observed at 1695 cm^−1^, attributed to the stretching of C=O, which is indicative of acetone formation. The presence of this band proves that the dehydrogenation path is promoted when more Co is present that is less acidic compared to the Fe‐rich sample, as observed by the emerging band for x=0.70 already after 10 s. Interestingly, this band only appears at early times of exposure and disappears on all samples almost after 1 min (Figure S11), which suggests that the acetone is either not stable on the surface or its formation is coverage dependent. The latter is supported by observing the spectra during desorption shown in Figure 2b, where we observed re‐appearing of the C=O band after 40 min of exposure to He for the Co‐containing samples. The time‐resolved spectra during desorption for each catalyst additionally showed a decreasing stability trend for the adsorbed 2‐propanol, as demonstrated in Figure S12. The insets of Figure S12 highlight the decrease of the stability consistent with less acidity for higher Co contents. The band at 1589 cm^−1^ is likely caused by the stretching of the C=C from propene formed through the dehydration path, although its corresponding band during 2‐propanol adsorption has been reported at ∼10–15 cm^−1^ lower wavenumbers on zirconates and titanates in the perovskite crystal structure.[^5d^, ^9^] Another possibility is a carboxyl vibration from acids like acetic acid as reported by Anke et al. at higher temperatures on CoFe_2_O_4_. ^[43]^

Other features were also observed at 1464 cm^−1^ and 1381 cm^−1^ which can be attributed to asymmetric and symmetric scissoring of ‐>CH_3_ and another small band at 1334 cm^−1^ related to scissoring of C−H bonds of 2‐propanol.[^5d^, ^43^] A very small band at 1242 cm^−1^ could also be detected from the scissoring of O−H bond of the gas‐phase or non‐dissociative adsorbed 2‐propanol.^[44]^ Two more bands were progressively developing at 1164 cm^−1^ and 1130 cm^−1^. The former is due to the stretching of the C−C bond, and the latter is related to both the rocking mode of ‐>CH_3_ and stretching of the C−O bond. It should be noted that the C−O bond can be formed by chemisorption of the 2‐propanol and the subsequent formation of 2‐isopropoxide. Therefore, its relative intensities compared to the C−C band could help to distinguish between the unstable adsorbed 2‐propanol (and/or other minor products of dehydrogenation and dehydration paths) and the more‐strongly adsorbed 2‐isopropoxide or other surface poisoning oxygenated species. The bands at 972 cm^−1^ and 949 cm^−1^ are most likely related to the isopropoxide^[45]^ and emanating from C−O stretching with hydrogen bonding to the surface^[46]^ in contact with ‐>OH groups.^[47]^ A summary of the bands′ assignment is given in Table S2. In conclusion, 2‐propanol adsorption DRIFTS showed that the selected catalysts x=0.00, x=0.10, x=0.25 and x=0.70 adsorb 2‐propanol and the position of the asymmetric stretching vibration shifts with Co incorporation into the materials. For x=0.70, consisting of three crystalline phases, two additional shoulders in the regime were observed. The formation of a C=O bond indicative for acetone formation was seen on all catalysts, although with a higher intensity for the less acidic Co containing catalysts, but the band intensity decreased after ∼1 min of exposure to 2‐propanol which indicates a coverage‐dependent formation of acetone since the band intensity increased again during desorption.

### Catalysis data evaluation procedure

**Figure 3:**
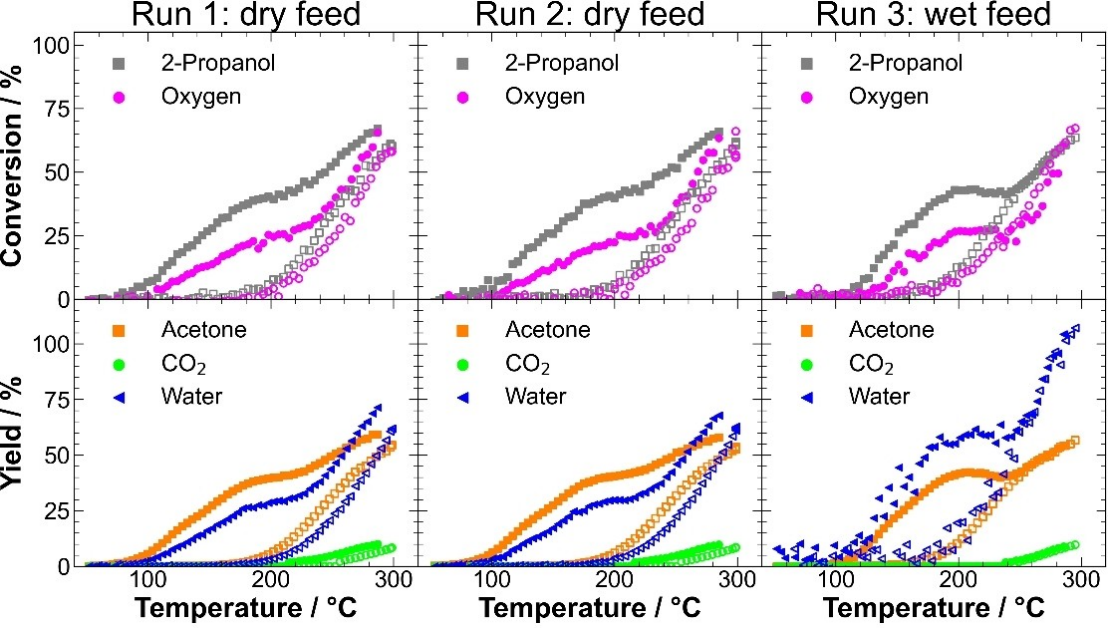
Exemplary dataset of x=0.25 obtained from gas‐phase 2‐propanol oxidation consisting of conversions of 2‐propanol and oxygen (top row) and product yields (bottom row) during three consecutive runs. Filled symbols show data points during heating. Empty characters show the behavior during cooling.

In Figure 3, an obtained example dataset is shown for x=0.25. The measurement protocol consisted of three consecutive transient experiments on the same catalyst material, two in a dry atmosphere and the third one in a wet atmosphere. The first two runs were performed in a 1 : 1 mixture of 2‐propanol and oxygen diluted with N_2_ without co‐feeding of water (dry feed). The second run was performed to check the restorability of the activity of the first run and stability of the catalysts as previously described by Anke and Falk et al.[^5b^, ^7^, ^43^, ^48^] The third run was performed with a 10‐fold excess of water compared to 2‐propanol and oxygen (wet feed) to build a bridge between gas‐phase and liquid‐phase catalysis. During initial catalyst activation by temperature‐programmed oxidation (TPO) at 300 °C in 10 % O_2_ for 2 h, CO_2_ formation was observed, which indicated the formation of carbonates or different adsorbed carbon species formed during storage in air (Figure S13). During the subsequent first run, conversions increased with the temperature proportionally for 2‐propanol and oxygen. The oxygen conversion indicates a main contribution of catalytic reactions compared to possible stochiometric reduction of the catalyst, in which only 2‐propanol would be consumed and no oxygen. Since both are consumed, a main contribution of catalysis compared to catalyst reduction can be concluded. A shoulder at 170 °C was observed, similar to CoFe_2_O_4_ catalysts.[^43^, ^48^] For 2‐propanol oxidation on LaFe_1−x_Co_x_O_3_, high‐temperature (HT) and low‐temperature (LT) channels were observed, comparable to previous works on Co_3_O_4_.[^5b^, ^10^] The LT channel persisted during heating to around 200 °C and featured selective oxidative dehydrogenation to acetone but is not stable. At different elevated temperatures, secondary product formation was observed, CO_2_ and propene were detected. After an isothermal dwell period of 1 h, the catalyst activity during cooling was significantly decreased, shown in decreased conversion and decreased acetone, CO_2,_ and propene yields. Activity loss during cooling was reported before in the literature. The loss was explained by partial stoichiometric reaction of 2‐propanol with the catalyst, namely the reduction of Co^3+^ to Co^2+^ in Co_3_O_4_,^[5b]^ acetate formation, and carbon deposition on CoFe_2_O_4_.^[43]^

During TPO between the runs, increased CO_2_ formation was observed, and the CO_2_ formation occurred at higher temperatures compared to the initial activation TPO, exemplarily shown for x=0.25 in Figure S13. The CO_2_ formation indicated buildup of carbonaceous compounds on the catalyst surface during the reaction and their burn‐off during TPO. Afterward, the LT activity channel was restored, due to the surface‘s reoxidation and the removal of carbonaceous compounds and carbon depositions from the surface.[^5b^, ^43^, ^48^] The activity of the second run, during both heating and cooling segments, matched the activity of the first run while the product distribution did not change as well. Indeed, the second run after TPO was performed on all samples to confirm structural stability of the catalysts and the possibility of restoring the activity of the initial freshly loaded catalysts. However, since both dry feed runs were reproducible and overlapped precisely, as shown in Figure S14 for all catalysts, only the first run was considered in the following for further analysis of the dry feed.

Furthermore, the activity of the catalysts upon water co‐feeding after a third TPO (Figure S13) showing CO_2_ formation overlapping with the second TPO was studied. Under wet feed conditions, the onset of the reaction was shifted to higher temperatures. In addition, an explicit maximum was observed at 200 °C instead of a broad shoulder in the same temperature range, as clarified in Figure S14e. Still, the main product was acetone as the product of the oxidative dehydrogenation path. In contrast to dry feed, the maximum conversion during wet cooling was not lowered compared to wet heating. While in the dry run, a pronounced activity decay was observed after the isothermal period at 300 °C, in the wet run, heating and cooling curves overlapped for T>250 °C. This might hint towards a temporary stabilizing effect of water on the catalyst surface structure, preventing either the reduction of active Co^3+^ species or the formation of carbonaceous deposits. The HT and LT channels and the instability of the latter remained present under wet conditions, which became evident by the decreased activity of the cooling curve compared to the heating curve at temperatures below 250 °C. In contrast to the heating curves, wet cooling showed higher activity and stability compared to dry cooling. Interestingly, the start of CO_2_ formation during heating was shifted to a higher reaction temperature at 240 °C. The before‐mentioned shift in the reaction onset to higher temperatures was in line with the result of adding water vapor to other oxidation reactions due to competitive adsorption of the reactants and water. At higher temperatures, a less severe effect of water in the feed was reported.[^10^, ^11^, ^12^, ^13^, ^14^, ^15^, ^16^, ^17^, ^18^]

To determine parameters for activity comparison, 2‐propanol conversion curves, 2‐propanol consumption rates, acetone formation rates, and CO_2_ formation rates were parameterized by fitting the dry and wet data. The heating and cooling curves were fitted with a 20^th^ order polynomial and a sigmoidal function, respectively. Figure S15a shows the heating and cooling curves of 2‐propanol conversion during the dry feed run, Figure S15b during the wet feed run. Parameters for comparing the catalysts, such as the temperatures to achieve 10 % and 50 % conversion (T_10_ and T_50_), were extracted from the fits. T_10_ during heating was used to describe the LT reaction channel, while T_10_ during cooling was used to describe the HT channel of the catalyst after the deactivation of the LT channel. T_50_ during cooling is used as another parameter to describe the HT channel.

Further parameters for comparison were derived from catalyst mass and BET surface area‐normalized rates at 150 °C during heating and 250 °C during cooling for the LT and HT channel, respectively. The methods of determination for these parameters are exemplarily shown for x=0.25 in Figure S15c and d. Both ways are presented to ensure the validity of trends derived from the parameter estimation. In summary, to characterize the LT channel, T_10_ and rates at 150 °C during heating were taken as activity parameters to compare the different catalysts. As HT channel parameters, T_10_ and T_50,_ and rates at 250 °C during cooling were considered. In the following, attempts are presented to correlate these parameters with the materials composition to determine the influence of Co content and phase mixtures.

### The effect of B‐cation substitution on dry feed selective 2‐propanol oxidation

**Figure 4:**
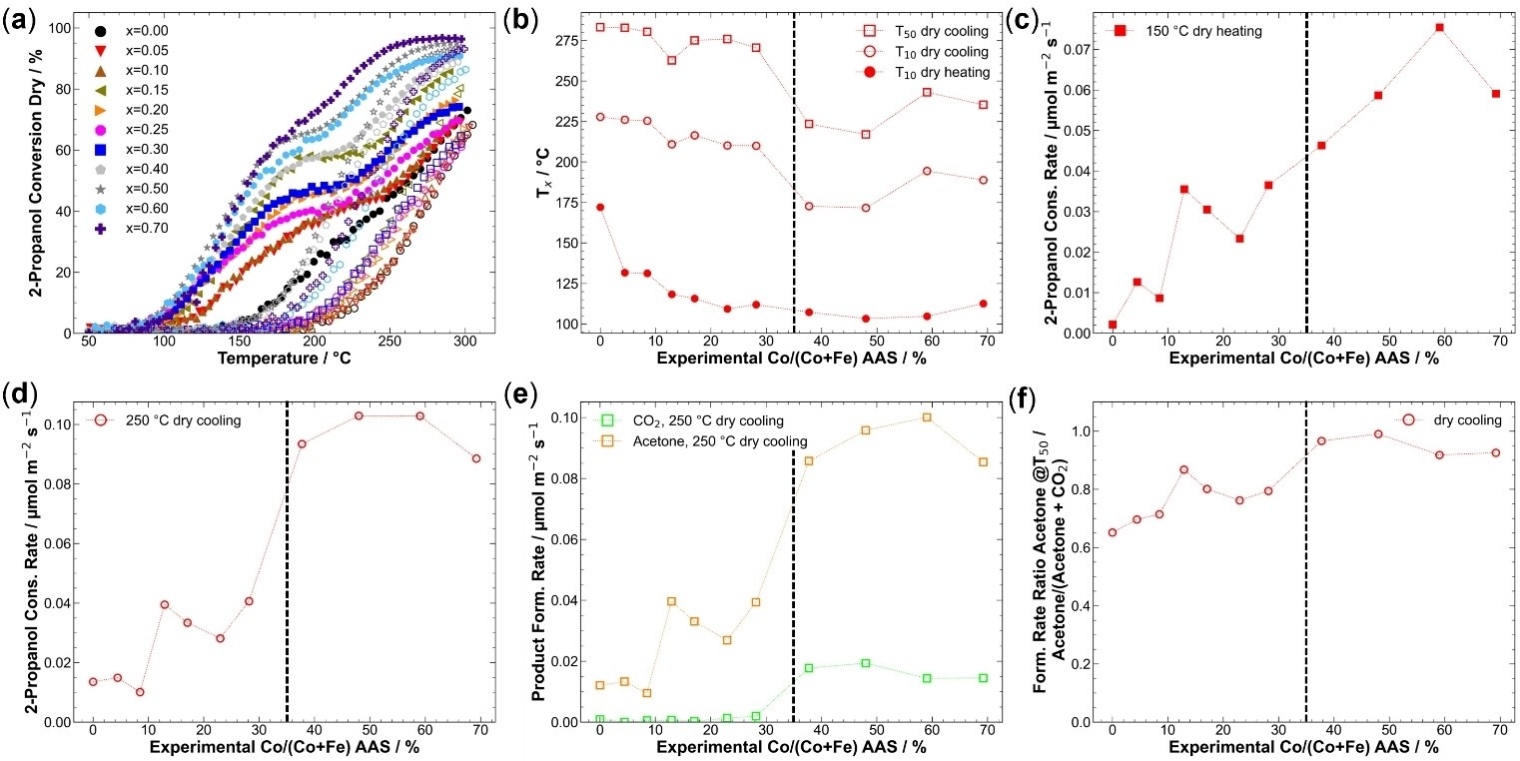
(a) 2‐Propanol conversions in dry feed. Solid and hollow symbols show data points during heating and during cooling, respectively. (b) T_10_ values during heating and cooling and T_50_ during cooling in dry feed plotted against the experimental Co content derived from AAS. (c) Surface‐area normalized 2‐propanol consumption rates at 150 °C during dry feed heating. (d) Surface‐area normalized 2‐propanol consumption rates at 250 °C during dry feed cooling. (e) Surface‐area normalized acetone and CO_2_ formation rates at 250 °C during cooling. (f) Surface‐area normalized formation ratio of acetone/(acetone+CO_2_) at T_50_ during cooling. Horizontal lines indicate the formation of the single orthorhombic perovskite phase at low Co contents and the three phase‐mixture at higher Co contents.

In Figure 4a, the 2‐propanol conversions of the catalysts in the dry feed are displayed. The conversions of oxygen are shown in Figure S16g. An s‐shaped profile was observed for all catalysts during heating. In general, the turning point during heating was shifted to lower temperatures with higher Co content, and the maximum conversion at 300 °C was increased with Co content. All catalysts showed the before‐mentioned HT and LT channel and deactivation of the latter during cooling compared to heating. Also, all curves during cooling featured a sigmoidal shape.

With the incorporation of only 5 % Co, a substantial increase in activity was observed as reported before for CO oxidation.^[19]^ The trend was observed in the LT channel as an initial sharp drop in T_10_ during heating in Figure 4b. A further more moderate T_10_ decrease with Co content in the catalysts was seen for all phase‐pure catalysts despite local activity maxima for x=0.15 and x=0.25, which was in line with higher CO oxidation activity and reducibility reported previously.^[19]^ In general, judging from T_10_ for phase‐pure samples in dry feed, an activity increase was observed with Co content. No jump in activity was recognized with the formation of secondary phases, but among the non‐phase‐pure catalysts, x=0.50 featured the lowest T_10_, while x=0.70 was the least active sample despite having the highest Co content.

T_10_ and T_50_ during cooling described the deactivated catalysts activity as parameters for the HT channel, and both showed a parallel evolution (see Figure 4b). For 0.00≤x≤0.30, a decrease with increasing Co content was detected. The strong activity boost of 5 % Co present during heating was not observed anymore, which might indicate a loss of the beneficial effect of Co^3+^ possibly due to reduction leading to similar performance like Co‐free LaFeO_3_, which is in line with the higher reducibility of Co compared to Fe.^[49]^ However, x=0.15 was found to be an evident local activity maximum in a volcano‐like fashion when considering only the phase‐pure samples with x<0.30. Since no clear deviation from the intended composition trend was seen for this sample in the previously presented thorough characterization, the reason remains unclear and might be related to the unique (electronic) structure effects of this specific perovskite composition. An extraordinary high activity of this perovskite composition was also reported before for CO oxidation.^[19]^ In that work, the position of the low‐temperature reduction peak during H_2_‐TPR indicated the highest reducibility for x=0.15 and a correlation between this increased redox activity and the catalytic properties was proposed. Upon the loss of phase purity, a jump to higher activity was observed for x≥0.40, with x=0.50 showing the highest activity suggesting a pronounced effect of the by‐phases on the HT channel activity, which was not observed for the LT channel.

2‐Propanol consumption rates in dry feed after normalization on exposed BET surface area are displayed in Figure S16c. In comparison to conversion data, differences in the relative reactivity were found after normalization. Rates at 150 °C during heating are shown in Figure 4c. In general, a rather steady increase of the 2‐propanol consumption rate with Co content in the catalyst is found, even though no linear correlation was discovered. The increase between x=0.00 and x=0.05 is not pronounced in this representation, and the volcano‐like behavior for the phase‐pure samples with a maximum at x=0.15 was observed, which was not as clearly seen for the LT channel in the T_10_ data. As discussed above, this may be tentatively attributed to higher reducibility of these samples.^[19]^ For phase‐mixed catalysts, a different trend compared to T_10_ was observed. Sample x=0.60 showed the highest activity instead of x=0.50, while x=0.70 still was less active than expected from its bulk Co content. Alike the T_10_ plot, no abrupt jump in catalytic properties with the presence of by‐phases is seen in the rate plot of the LT channel.

The reaction rate at 250 °C during cooling characterizes the HT activity channel and is shown in Figure 4d. Within the phase‐pure perovskite samples, x<0.40, the volcano‐shape with a maximum at x=0.15 from the LT channel was again confirmed in the HT channel. However, the non‐phase‐pure catalysts showed a pronounced and abrupt activity increase compared to phase‐pure ones as observed before for T_50_ during cooling. The relative activity within the high Co content samples is different compared to the LT channel and the rates for all four catalysts were similar during cooling at 250 °C, with x=0.50 and x=0.60 showing equal and the highest activities. Also, the activity decay for x=0.70 was confirmed.

Product formation rates at 250 °C indicated a jump in CO_2_ formation with the appearance of secondary phases, as is shown in Figure 4e. For acetone formation rates, the same trends as in 2‐propanol conversion were seen for all catalysts. When comparing the selectivity in terms of acetone/(acetone+CO_2_) product ratio at T_50_ during cooling, as shown in Figure 4f, it becomes evident that despite the jump in CO_2_ formation, the selectivity increases rather steadily with Co content even in the presence of by‐phases. The local activity maximum at x=0.15 is clearly reflected in a selectivity maximum. The catalyst x=0.50 showed the highest selectivity and selectively catalyzed the formation of acetone at its T_50_.

Product yields for all catalysts are shown in Figure S17. For all catalysts, acetone formed via the oxidative dehydrogenation pathway was the main product of the selective oxidation reaction accompanied by the coupled product water. On the other hand, the most prominent undesired oxidation product was CO_2_. Formation of propene from dehydration in dry feed indicates a more acidic surface of Fe‐rich catalysts as x=0.00, x=0.05, and x=0.10 show comparably high yields of propene, which is in line with expectations raised by the literature.[^23^, ^25^] Also, the results agreed with a higher content of B‐cations on the materials’ surface with low Co‐contents found by XPS. This indicates a more acidic surface caused by Fe‐excess on the surface. For all other catalysts, the maximum propene yield was less than 1 %, the onset temperature shifted to higher temperatures with increasing Co content. Interestingly, the propene formation was not as affected by the deactivation of the catalyst, the yields during heating and cooling were comparable. Other detected products were diisopropyl ether and acetic acid, both of which showing low yields. Ether formation was detected for catalysts with x<0.40 while acid formation was observed in traces for x=0.00, x=0.05, x=0.10, x=0.15 and x=0.30.

Summarizing the dry‐feed activity, the Co‐free sample showed poor activity, whereas Co‐incorporation played a dominant role in 2‐propanol oxidation activity in the LT channel. In the HT channel, not only the nominal composition but also phase composition was decisive. In the presence of rhombohedral perovskite and spinel, there was an extra activity boost. Within the phase‐pure orthorhombic perovskites, a volcano‐like behavior was observed with a maximum of 15 % Co, which might be superimposed to the composition effect in both channels. A similar non‐linear activity increase was reported before in CO oxidation and exhaust gas decomposition on LaFe_x_Co_1−x_O_3_,[^19^, ^50^] Co‐doped NiO in CO oxidation,^[51]^ and LaFeO_3_ with Co and Sr doping.^[23]^ For all catalysts, acetone was the main product. Fe‐rich catalysts showed the highest propene yields, which indicates that Fe is the more Lewis‐acidic site compared to Co.

### The effect of water vapor on the 2‐propanol oxidation

**Figure 5:**
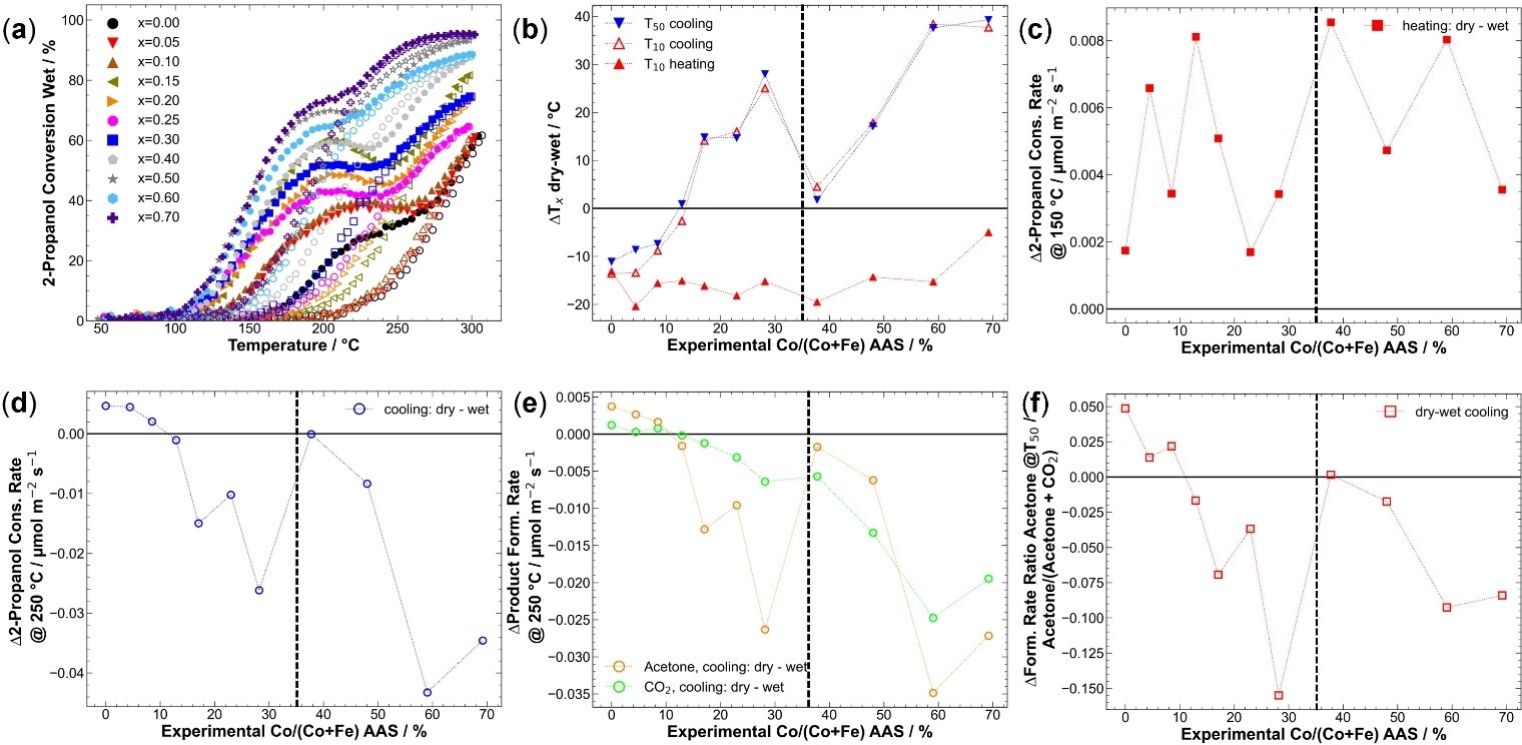
(a) 2‐Propanol conversions in wet feed. Solid and hollow symbols show data points during heating and during cooling, respectively. (b) Differences between T_10_ during heating cooling and T_50_ during cooling for T_x_ dry and T_x_ wet differences (Δ=dry‐wet) plotted against the experimental Co content derived from AAS. (c) Differences between surface‐area normalized 2‐propanol consumption rate at 150 °C during heating. (d) Surface‐area normalized 2‐propanol consumption rate differences at 250 °C during cooling. (e) Differences in surface‐area normalized acetone and CO_2_ formation rates at 250 °C during cooling. (f) The difference in the ratio of acetone/(acetone+CO_2_) at T_50_ during cooling. Horizontal lines indicate the formation of the single orthorhombic perovskite phase at low Co contents and the three phase‐mixture at higher Co contents.

Wet feed 2‐propanol conversions of the third run are shown in Figure 5a; wet feed oxygen conversions are shown in Figure S16h. The curves despite x=0.00, x=0.30, x=0.70 showed a clear local maximum during heating for the LT channel, in contrast to dry feed runs, where only a shoulder was observed. Also, under wet conditions, the deactivation of the LT channel was observed like in the dry feed for all catalysts, and the cooling curve showed a sigmoidal shape without maxima. Compared to the dry runs, the difference between heating and cooling was decreased, which was reflected in cooling curves shifted to lower temperatures, especially for Co‐rich materials. In the HT channel, x=0.40 and x=0.50 showed even an activation and higher activity during cooling than heating until 235 °C and 210 °C indicating a promoting effect of water on the HT channel.

Because of similar curve shapes in dry and wet feed, a comparison based on the previously presented parameters shown in Figure 4 for dry feed is also valid in wet feed. In Figure S18, the mentioned parameters are displayed for dry and wet feed. In the following paragraphs, the differences between dry and wet conditions will be discussed based on the differences between the parameters.

Conversion onset temperature, where the first conversion was observed, during heating was increased for all catalysts from below 100 °C in dry feed to over 100 °C in wet feed. From individual T_10_ parameters, the lowest T_10_ in wet feed was 118 °C for x=0.50 and x=0.70. A parametrized indicator for this rise is the ΔT_10_ value between dry and wet conditions, as shown in Figure 5b. A ΔT<0 indicates higher temperatures to reach the same conversion and thereby lower activity. As indicated by T_10_ during heating, the water addition into the feed increased the LT channel onset. The onset of the reaction shifted by at least 12 °C for all catalysts except x=0.70, for which the onset was only shifted by 5 °C. Also, under wet conditions, the LT boost of only 5 % Co was pronounced, as shown in Figure S18a. The activity loss upon water co‐feeding can be explained by the competitive pre‐adsorption of water on the surface and blocking of active sites, as reported in the literature.^[18]^ The water adsorption is considered to take place at the acidic centers of the catalyst surface.^[6]^ Also, in methane oxidation, an adverse effect of water on conversion at lower temperatures was observed on La_1−x_Sr_x_FeO_3_ catalysts.^[15]^ Otherwise, no evident influence of the Co content on the change in LT activity was observed suggesting that above 100 °C, the competitive adsorption does not play a substantial role anymore. Thus, at higher temperatures, the detrimental effect of water addition was less pronounced, as observed also in the literature for other oxidation reactions.[^15^, ^18^]

From Figure S18a, a less‐pronounced HT channel boost of x=0.15 in wet feed compared to dry feed can be concluded, along with no major effect of the incorporation of 5 % Co as observed in the dry feed. Also, a clear jump in the HT channel between phase‐pure and non‐phase‐pure materials was not observed as strongly as in dry conditions. ΔT_50_ and ΔT_10_ during cooling characterized the HT activity difference, which revealed similar behaviors (Figure S18a). For Fe‐rich catalysts with x<0.15, the effect of water on the HT‐channel was found to decrease the catalytic activity and higher temperatures were needed for 10 % or 50 % conversion as is evident from the plot of ΔT_x_ (dry‐wet) in Figure 5b. This effect, however, decreased with increasing Co content and the negative ΔT_x_ vanished at x=0.15 for the HT channel, while it persisted in the LT channel. An activity increasing impact of water‐addition on the HT activity was observed for x>0.15 with positive Δ‐values for the HT channel. A local maximum was reached at x=0.30, a significant drop for x=0.40 was observed, which indicated a different interaction of different perovskite structures and spinels upon water addition into the feed. Upon further increase of the Co content and phase segregation, the positive effect of water on the HT channel was even more pronounced. A similar but higher increase of the HT channel activity was previously reported for Co_3_O_4_ spinel catalysts.^[10]^ In addition, a more pronounced decrease in the activity of LaFeO_3_ was reported compared to LaCoO_3_ in the oxidation of propane and NO upon co‐feeding water.^[18]^

2‐Propanol consumption rates in wet feed after normalization on exposed BET surface area are displayed in Figure S16b. To further evaluate activity differences between dry and wet feed, rate differences at 150 °C during heating (HT channel) and 250 °C (LT channel) were compared based on their difference dry‐wet (Δ). Positive values indicated higher rates of dry runs and negative values higher rates during wet reactions. Differences at 150 °C as representatives of the LT channel are shown in Figure 5c. Individual dry and wet values in Figure S18b show that the general trend of the dry feed runs was retained for wet runs with a volcano‐shaped curve for the phase‐pure samples with x<0.40 with a maximum at x=0.15. The differences were comparably low between the dry and wet feed, but for all catalysts, the activity in dry feed was slightly higher compared to the wet feed in line with the lower T_10_ during heating (Figure S18a). There was no systematic effect with Co content on the difference between reaction rates at 150 °C during heating. Like the ΔT_10_ heating values the rate differences scatter around an average, indicating a negative effect of water.

On the other hand, water addition strongly affected the HT channel characterized by the 2‐propanol consumption rates at 250 °C during cooling, as also observed for ΔT_50_. The difference curve is plotted in Figure 5d, while the individual dry and wet rates are shown in Figure S18c. These indicate a rather constant rate increase with Co incorporation among nearly the whole composition range including the non‐phase‐pure catalysts until a maximum at x=0.60. For phase‐pure orthorhombic perovskites in wet feed, the superimposed volcano‐shaped curve was less clear than in dry conditions and the local maximum was shifted to x=0.20.

In the rate difference plot (Figure 5d), a decrease in the difference with the Co content was observed, which indicated a beneficial interaction of 2‐propanol with the Co‐richer surfaces under wet conditions. As seen in the ΔT_10_ and ΔT_50_ plots for the HT channel, water addition into the feed was detrimental for x<0.15 as indicated by a positive value for the rate difference, with nearly no effect for x=0.15, while for higher Co contents, a beneficial and promoting effect was observed with negative rate Δ. For the catalysts containing three crystalline phases, the difference between heating and cooling was non‐observable for x=0.40, but with increasing Co content, the beneficial effect of water was also established for these catalysts. The same trend was observed for acetone formation, as shown in Figure 5e. On the other hand, for x≤0.20, the CO_2_ formation was not affected by the co‐feeding of water, while for increased Co contents in the catalyst, the increase in CO_2_ formation under wet conditions nearly showed a linear trend up to x=0.60. Based on the product ratio at T_50_ during cooling, as shown for dry and wet feed in Figure S18e and as a difference curve in Figure 5f, for x<0.15, a slight loss in selectivity towards selective oxidation in wet feed was found for Fe‐rich catalysts, whereas for Co‐rich catalysts, the addition of water was beneficial towards a higher ratio of acetone compared to CO_2_.

The product yields are shown in Figure S17. Like in the dry feed, acetone was the main product for all catalysts accompanied by the couple product water, formed through the oxidative dehydrogenation pathway, while CO_2_ was the main secondary product. The onset of propene yield shifted to higher temperatures with increasing Co content, starting from 235 °C (x=0.00) in wet feed instead of 185 °C in dry feed. Also, the maximum yield of propene was decreased for all catalysts and decreased from 3.6 % to 1.6 % (x=0.00). Only catalysts with x<0.20 showed the formation of propene in the wet feed, which indicated a higher suppression of water co‐feeding on the dehydration pathways compared to oxidative dehydrogenation (acetone) and total oxidation (CO_2_). This was in line with the expectation, as the acidic sites for dehydration are competitively adsorbing water and 2‐propanol and therefore are partially blocked, whereas the basic sites are still available to perform oxidative dehydrogenation. Also, the formation of diisopropyl ether and acetic acid was influenced by the co‐feed of water. While acetic acid was not observed in wet conditions anymore, the onset of ether formation shifted to higher temperatures by ∼20 °C, and the maximum yield decreased from 0.6 % to 0.4 %. Ether formation was observed in dry and wet feed for x<0.30.

Since, depending on the Co content, wet feed data showed a shift of the HT channel to lower temperatures at the same conversion, a stabilizing effect of water on the HT channel is postulated that might be related to a slower deactivation of the surface by reduction and/or formation of carbonaceous deposits. Therefore, steady‐state experiments under dry and wet conditions were performed for x=0.00 and x=0.25 to study the effect of Co addition in the regime of phase‐pure perovskites, which are shown in Figure S19 and Figure S20. For x=0.00, conversion dropped from 52 % to 45 % within 3 h in dry feed, while under wet conditions, the conversion was stable in the range of 40 % from the beginning of the isothermal period. In contrast, in dry feed for x=0.25, an initial activity decay within 2.5 h in the dry feed from 51 % to 23 % was observed. Under wet feed conditions, the deactivation took place within 4 h from 54 % to 14 % conversion. Afterward, the conversion remained constant in both cases in a regime where the activity of the HT‐channel during cooling in the transient experiment was observed. This indicates that the low‐temperature activity channel is deactivated and can only be seen in a cyclic protocol and restored after another TPO. These experiments supported the argument of the stabilizing effect of water, which leads to a slower deactivation of the Co‐containing catalysts, which affects the cooling data in the transient experiments after the isothermal period at 300 °C. Also, the experiments showed lower steady‐state activity in wet feed, as also reported in the literature.[^10^, ^15^] Potential reasons for the slower deactivation might be suppressed or slower reduction of Co^3+^ to Co^2+^ due to water adsorption on Co^3+^ or different amounts of carbonaceous species deposition on the catalysts, both shown as deactivation mechanisms for spinel‐based catalysts.[^5b^, ^43^] Another possible explanation is the interaction of water molecules with oxygen vacancies. Competitive adsorption of water and oxygen on anion vacancies was reported in the literature and also a hypothesis about a decrease of oxygen vacancies on the catalyst was made.^[18]^ However, also a formation of oxygen vacancies in wet feed is possible.

In summary, a detrimental effect of water on the LT channel was seen upon co‐feeding water and the onset of the reaction in the LT channel shifted to higher temperatures due to competitive adsorption of water and 2‐propanol, while the rates decreased. At the same time, there was a two‐fold effect on the HT channel. First, there is an activity decrease for Fe‐rich catalysts under wet conditions compared to dry conditions, whereas Co‐rich catalysts were more active with water present in the feed in the HT state after deactivation of the LT channel showing how feed and catalyst composition interact for this promoting effect. A likely explanation is that the deactivation of catalysts proceeded slower under the co‐feed of water if at least 10 % Co is incorporated into the structure, explaining the detrimental effect of water on the HT channel activity for Co‐poor materials.

### Compositional and structural analysis of spent catalysts

In Figure S21, compositional analysis of the surface/bulk B‐cation distribution is displayed after catalysis. For the analysis, spent samples were taken from the quartz reactor after cooling down of the wet feed run, stored in air, and characterized. The overall composition trends after reaction (post‐catalysis) matched the trends of as‐prepared samples before catalysis. As seen on the fresh samples, the catalysts were deficient in Co on the B site on the surface, as derived from XPS, compared with the bulk, as determined by STEM‐EDX. In Figure S22a, difference plots of the Co/(Co+Fe) ratio from STEM‐EDX and XPS are displayed for direct comparison of the two states, i. e., before and after catalysis. The values are statistically spread around the baseline of 0, indicating no general change among the sample series and no preferred diffusion of Co or Fe to the surface or the bulk during the reaction.

Judging based on the redox‐active cation distribution Co/(Co+Fe), the structure remained largely intact during the reactions, as no indications for systematic compositional changes of the ratio were observed on the surface or in the bulk. XRD after steady‐state analysis (shown in Figure S23) strengthened the argument of a retained perovskite structure after catalysis. The perovskite structure remained intact for both cases after two cycles of 24 h reaction time. The only prominent change is the appearance of an increased background in the region between 20° and 35°, that can be attributed to the contribution of quartz wool, used as catalytic bed support. In a previous study on CO oxidation, no bulk changes of LaCoO_3_ were detected by XRD as well.^[3e]^ After catalysis, no changes were observed in the ratio of redox‐active cations and the bulk‐structure was maintained. The combination of both findings indicates that the characterization of as‐prepared catalyst materials may be used as a first approximation for knowledge‐based synthesis of perovskite‐type oxidation catalysts at temperatures up to 300 °C, since bulk properties and surface composition remain largely unchanged after catalysis at relatively low temperature.

**Figure 6:**
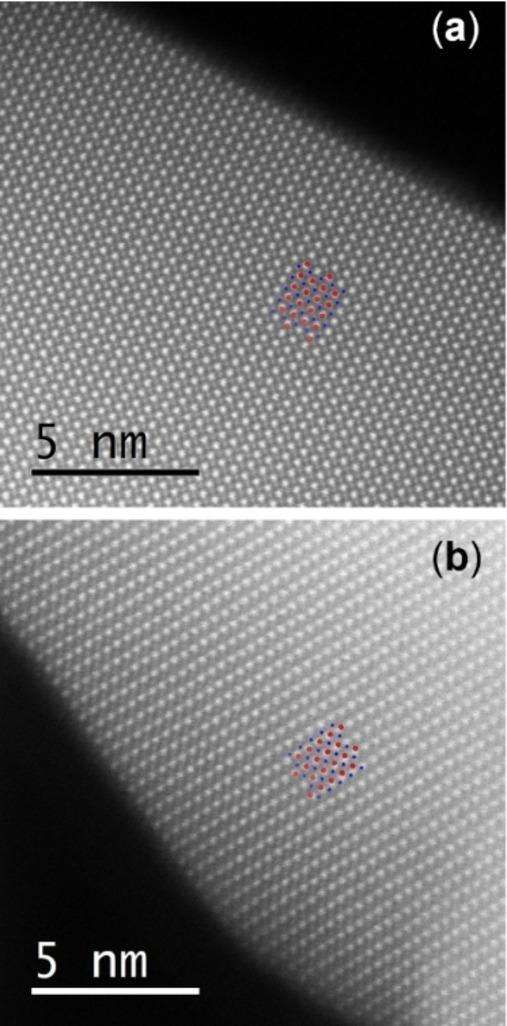
(a) HAADF‐STEM micrograph of x=0.30 in the as‐prepared state. (b) HAADF‐STEM micrograph of x=0.30 in the post‐catalysis state. The dots on the lattice of both subfigures show the LaFeO_3_ structure along the [110] direction, Fe ions in blue and La ions in red.

In addition, in High Angular Dark Field (HAADF) STEM analysis of x=0.30 before and after the cyclic catalytic protocol, as shown in Figure 6a in the as‐prepared state and in Figure 6b post‐catalysis, the orthorhombic perovskite structure remained intact, as indicated by the exemplary crystal structure of the LaFeO_3_ structure along the [110] direction in both subfigures. This supports the argument of an intact perovskite structure after catalysis from a microscopic point of view as well. Altogether, structural characterization after the reaction indicates a stable bulk of the perovskite catalysts even under reaction conditions. However, the surface can be considered as the dynamic part during the reaction, which is supported by the compositional analysis comparing the as‐prepared and post‐catalysis states.

After catalysis, the trend in B‐cation to A‐cation distribution with B‐cation enrichment remained present, as displayed in Figure S21b. The differences of the STEM‐EDX data still indicate a largely unchanged La‐enrichment in the bulk of the materials for 0.00≤x≤0.70. With some deviating data points and an increased error bar compared to one individual measurement, nearly all Δ values of the XPS data comparing those measurements before and after catalysis indicate a decrease in the ratio of B‐ to A‐cations in regions near the surface even though the bulk structure remained unchanged. Effectively, for the phase‐pure catalysts up to x=0.30 La enrichment on the surface decreases with increasing Co content until the bulk value is reached. For the catalyst materials with x≥0.30 after catalysis, no La surface enrichment is observed anymore (see also Figure S22b). Apparently, migration of La to the catalyst‘s surface during the reaction or diffusion of Co and Fe into the bulk structure occurred during the reaction. Surface changes in perovskites were also reported in the literature on catalysis and temperature treatment in an oxidizing atmosphere.[^26d^, ^52^] For SrTiO_3_, an A‐cation enrichment on the surface was found upon heat treatment, shown by an increase of Sr/(Sr+Ti) ratio after O_2_ treatment.^[26a]^ The same applied for BaZrO_3_ above 500 °C.^[53]^

TEM micrographs after the reaction (Figure S8 and Figure S9, also in comparison with micrographs before the reaction) can be seen as examples for surface dynamics during catalysis, such as surface reconstruction, including surface roughening, surface amorphization, or faceting. The reconstruction to more faceted surfaces was observed in literature upon heat treatment, but at temperatures in the range of 750 °C.^[53]^ However, a detailed and statistical analysis of the changes in the STEM images was beyond the scope of the present investigation.

To sum up, according to the comparison of fresh and spent catalysts, the bulk structure remained intact after catalysis. However, the ratio of A‐cations to B‐cations on the surface, determined by XPS, was changed. Despite the bulk properties of the catalysts seem to be unaffected, changes during catalysis are present at the surface and require operando studies of the catalytic surface, for example in electron microscopy or near ambient pressure X‐ray photoelectron spectroscopy (NAP‐XPS).^[54]^

### Operando NAP‐XPS: Effect of water on x=0.25

**Figure 7:**
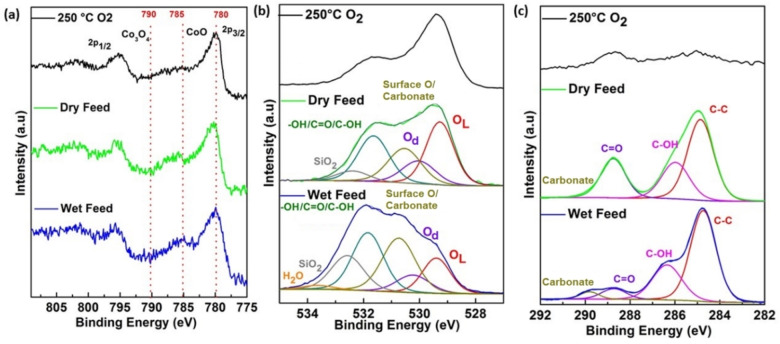
Operando NAP‐XPS during 2‐propanol oxidation under steady state conditions (a) Co 2p spectra (hν=930 eV; KE=150 eV). (b) Deconvoluted O 1s spectra (hν=680 eV; KE=150 eV). (c) Deconvoluted C 1s spectra (hν=430 eV; KE=150 eV). Top, middle, and bottom panel show: the oxidative pretreatment, dry and wet feed conditions.

The effect of water vapor on the 2‐propanol oxidation was studied exemplarily for the LaFe_0.75_Co_0.25_O_3_ (x=0.25) sample, using NAP‐XPS, under steady‐state conditions, which is comparable to the measurements shown in Figure S20. This technique is potentially able to provide insight into cobalt oxidation states, oxygen defects and adsorbed species at the catalyst surface. Therefore, NAP‐XPS upon pretreatment of LaFe_0.75_Co_0.25_O_3_ (x=0.25) up to 250 °C in 0.25 mbar of oxygen was investigated, followed by an investigation in the dry and wet reaction mixtures (total pressure 0.25 mbar) at 200 °C. For each reaction mixture, a fresh catalyst was used. The resulting spectra are shown in Figure 7.

Focus of the analysis was the Co, as this element boosts the catalytic performance and is the redox‐active material in this system, and the selected catalyst has a relative high Co content among the phase‐pure perovskite catalysts. The corresponding Co 2p spectra are shown in Figure 7a and exhibited a main Co 2p_3/2_ signal around a binding energy (BE) of 780 eV together with a satellite structure. The latter is sensitive to the Co oxidation state as the characteristic satellites for Co_3_O_4_ and CoO are around 790 eV and 785 eV BE, respectively.^[55]^ For pretreatment in O_2_ the broad satellite structure between 790 eV and 783 eV BE indicated a partial reduction of Co^3+^ to Co^2+^. In contrast, at reaction conditions for both wet and dry feed a single satellite feature around a BE of 783 eV meant a fully reduction to Co^2+^. This is also confirmed by a linear combination fitting using the peak shapes of Co_3_O_4_, CoO and Co (Figure S24) reported by Biesinger et al.^[55]^ Close inspection of the peak shape in case of the wet feed revealed some additional peak contribution at 783 eV BE in addition to a slightly broader Co 2p_3/2_ signal. This may suggest an additional formation of Co hydroxide species which should have a broad satellite around this region.^[56]^

In the O 1s region of dry and wet feed, a broad signal with overlapping components was detectable. A possible deconvolution is provided in Figure 7b. The components of the substrate can be attributed to oxygen lattice (O_L_) at BE=529.3 eV and to oxygen related to defects (O_d_) at BE=530 eV.^[57]^ The defective type oxygen can be understood as a missing oxygen atom, whereas the missing additional charge is compensated by the surrounding oxygen atoms resulting in a peak shift compared the nominal lattice BE of O_L_. An alternative explanation of this peak at BE=530 eV could be a contribution of common impurities like molybdates that were found in small amounts on the samples. The peak at BE=530.7 eV can be attributed to adsorbed and surface oxygen species (surface O).^[57]^ In addition, around this binding energy there are also carbonates that were detected in the case of the wet feed in the C 1s spectrum (see below). The signal around BE=531.8 eV corresponds to carbonyl/alcoholic O as well as to hydroxide formation and could not be resolved into single components. Furthermore, adsorbed water (BE=533.8 eV) and SiO_2_ contaminations at BE=532.6 eV can be detected. The main difference between dry and wet feed is an increased intensity of signals related to the hydroxide formation and the carbonate adsorbates. The first one is in line with the presence of water in the wet feed.

C 1s spectra in Figure 7c after pretreatment indicated no major adsorbed carbon, but under dry conditions, contributions of C=O (BE=288.7 eV), C−OH (BE=285.9 eV) and C−C (BE=284.8 eV) were observed. The signals of C−OH and C−C could arise from adsorbed 2‐propanol or some fragments of it. On the other hand, the appearance of C=O can be explained by intermediates of the reaction or even not yet desorbed acetone. Under wet conditions, an additional signal at a BE of 289.8 eV appeared that could be interpreted as carbonate formation. In addition, the carbonyl signal was lowered in intensity and shifted towards lower BE by 0.2 eV. The C−OH signal shifted by 0.3 eV to higher BE. Both shifts may be explained by a different interaction of the adsorbed species with the dry and wet surface.

In summary, a reduction from Co^3+^ to Co^2+^ was observed in the reaction mixture of gases. According to the sample history with a pre‐annealing step and with a temperature of 200 °C, the measurements were performed in the deactivated part of the HT channel. These conditions are in line with the observed reduction of Co^3+^ to Co^2+^. It is worth mentioning that during these measurements a formation of acetone, CO_2_ and water was observed via mass spectrometry. The main differences from comparison between dry and wet feed were increased hydroxide and carbonate signals visible in the O 1s spectra. The high amount of adsorbed carbon species on the surface may also explain the deactivation of the catalyst by a blocking of the active sites.

Further operando studies on the selected catalyst are planned and under execution to fully understand the role of water in the reaction mixture and the individual transition metals.

## Conclusion

LaFe_1−x_Co_x_O_3_ catalysts with an orthorhombic crystal structure were synthesized phase‐pure up to x=0.30. For x>0.30, a three‐phase mixture was found consisting of orthorhombic perovskite and additional rhombohedral perovskite and minor amounts of a spinel structure. By Rietveld refinement, a high content of Co in the spinel was observed. From Mössbauer spectroscopy, the weak ferromagnetism, and typical hyperfine parameters of Fe^3+^ ions in the perovskite phase were confirmed, while magnetometry data indicated the preservation of the macroscopic stoichiometry throughout the sample series, with only minute parasitic phase content. The materials are Co deficient on the B‐site, both at the surface and in the bulk, particularly at high Co contents, i. e., x≥0.30. The adsorption of 2‐propanol under dry conditions was studied for selected catalysts in a DRIFTS setup during adsorption and desorption of 2‐propanol and showed an immediate formation of an acetone band after short exposure to the reaction mixture, but a disappearance of the band at higher surface concentrations of 2‐propanol. Also, a higher acidity of Fe‐rich surfaces was confirmed during product analysis of the catalytic reaction in a flow‐setup. LaFe_1−x_Co_x_O_3_ can be considered as active and selective catalysts for gas‐phase 2‐propanol oxidation in a catalytic flow‐reactor. The reaction offers two pathways, a more active unstable pathway (LT‐channel) and a less active, but stable pathway (HT‐channel). The transition between the two channels can be ascribed to changes in transition metal oxidation state and deposition of carbonaceous compounds on the surface due to stoichiometric reaction instead of catalytic reaction and can be restored by an oxidative treatment in between the oxidation runs. In general, higher Co contents in the perovskites increase the reactivity of the catalysts with the most pronounced boost between x=0.00 and x=0.05. In HT‐activity, a volcano‐like behavior was observed among the phase‐pure samples with a maximum at x=0.15 which is in line with the lowest TPR peak position presented in a previous study.^[19]^ With the evolution of the two secondary phases, another pronounced activity jump was observed. The addition of water into the feed had a negative impact on the LT channel over the whole composition range. For the HT channel, several effects were observed among the phase‐pure samples. For catalysts up to x=0.10, a negative effect of the water co‐feed was seen. For x=0.15, nearly no change was observed, while for x>0.15 there was a positive effect of water. The positive effect of water in the feed might be explained by water adsorption on Co^3+^ and therefore preventing the active Co^3+^ sites from fast reduction and/or being deactivated quickly due to coking. NAP‐XPS showed an increase in hydroxide content on the surface upon the introduction of water into the feed. Furthermore, the reduction of Co^3+^ during the reaction cycle in dry and wet feed was shown, which might point toward an only temporary stabilization of the surface in the wet feed in the transient experiment. The comparison of as‐prepared samples before catalysis and spent‐sample characterization in TEM(‐EDX) and XPS indicated that the initial Co deficiency was not changed after catalysis for phase‐pure catalysts. The bulk of the materials generally shows an A‐cation enrichment, which is not changed after catalysis. Pronounced B‐cation enrichment on the surface is observed that is decreased after the reaction. For the catalysts also featuring secondary phases, B‐cation surface enrichment, when compared with bulk value, is significantly less pronounced and vanishes after catalysis. Generally, no large‐scale structural changes of nanoparticles like particle size, shape and bulk composition were observed between the samples before and after catalysis, justifying a synthesis approach targeting the rational design of low‐temperature oxidation catalysts. In‐depth kinetic analysis to fully understand the effect of water in the reaction mixture and the impact on individual transition metals, is currently being done in our consortium for the conditions and materials elaborated in this work and will be reported in an upcoming research paper.

## Experimental Section

### Raw materials

For the synthesis of the investigated catalysts, commercially available reagents were used without additional purification: lanthanum(III) nitrate hexahydrate (99.9 % La, abcr GmbH, Karlsruhe, Germany), iron(III) nitrate nonahydrate (≥98 %, Sigma‐Aldrich GmbH, Karlsruhe, Germany), cobalt(II) nitrate hexahydrate (≥98 %, Carl Roth GmbH, Karlsruhe, Germany), sodium hydroxide (98.5 %, Carl Roth GmbH, Karlsruhe, Germany), and sodium carbonate (≥99.5 %, VWR International GmbH, Darmstadt, Germany).

### Synthesis and sample preparation

The synthesis via co‐precipitation included preparing metal salt stock solutions with a total ionic concentration M^x+^ of 0.8 mol L^−1^ with the general composition La^3+^/Fe^3+^/Co^2+^=1 : 1−x : x. The value of x was varied in the range between 0.00 and 0.70. The precipitation agent was a solution consisting of 1.2 M NaOH and 0.18 M Na_2_CO_3_.

The syntheses were performed in an automated OptiMax 1001 (Mettler Toledo GmbH, Greifensee, Switzerland) synthesis workstation. The setup consists of a single‐walled glass reactor fixed inside a solid‐state thermostat for accurate temperature control. During precipitation and aging steps, N_2_ flow was employed, and the 100 mL prefill volume of the reactor was purged with N_2_ for 30 min. The co‐precipitation experiments have been performed isothermally at 10 °C and a constant pH of 9.5. A universal control box equipped with a precision balance allowed gravimetric dosing of the metal salt solutions of 75 g in 36 min. Control over the pH was achieved by simultaneous computer‐controlled dosing of the metal salt solution and the precipitation agent via two ProMinent gamma/L metering pumps. The pH was monitored and adjusted using an InLab Semi‐Micro‐L electrode during each experiment. A pitched blade impeller rotating at a constant speed of 300 rpm was used to avoid concentration and temperature gradients. After the precipitation was finished, an aging step at 10 °C for 60 min was performed. After aging, the precipitate was isolated by centrifugation (6000 rpm, 2 min) and washed with deionized water until the conductivity of the supernatant was below 0.1 mS cm^−1^ in two consecutive runs. Afterward, the samples were dried in static air at 80 °C for 12 h. Finally, the precursors were calcined at 800 °C for 3 h (*β*=2 °C min^−1^) in stagnant air in a muffle furnace (B150, Nabertherm, Lilienthal, Germany). The calcined samples were characterized as powders and pressed with a hydraulic press by Perkin‐Elmer (5 t, 2 min, Überlingen, Germany), pestled, and sieved with stainless steel sieves ATECHNIK (ISO 3310‐1, Leinburg, Germany).

### Catalyst characterization

Fe and Co contents in the precursors were determined by atomic absorption spectroscopy (M‐Series, Thermo Electron Corporation, Waltham, Massachusetts, United States of America).

Thermogravimetric measurements (TG) were performed in a NETZSCH STA 449F3 thermal analyzer (NETZSCH‐Gerätebau GmbH, Selb, Germany). For TG measurements, 50 mg of precursor material was heated in a corundum crucible in 21 % O_2_ in Ar from 30 °C to 1000 °C with a linear heating rate of 5 °C min^−1^.

N_2_ adsorption‐desorption experiments were performed with a NOVA3000e setup (Quantachrome Instruments, Boynton Beach, Florida, United States of America) at −196 °C after degassing the samples at 80 °C for 2 h in a vacuum. BET (Brunauer Emmet Teller) surface areas were calculated from *p*/*p*_0_ data between 0.05 and 0.3. Total pore volumes were determined at *p*/*p*_0_=0.99.

Powder XRD patterns in the 2θ range from 5 ° to 90 ° were recorded on a Bruker D8 Advance (Bruker, Billerica, Massachusetts, USA) diffractometer in Bragg–Brentano geometry using a position‐sensitive LYNXEYE detector (Ni‐filtered CuK_α_ radiation, Bruker, Billerica, Massachusetts, USA) applying a counting time of 0.3 s and step size of 0.018. Samples were mounted using dispersion in ethanol on a glass disc inserted in a round PMMA holder. The latter was subject to gentle rotation during scanning after removing the ethanol by drying. For structure analysis and calculation of lattice parameters, Rietveld refinement [44] was applied using the TOPAS software (Bruker, Billerica, Massachusetts, USA).

X‐ray photoelectron spectroscopy (XPS) measurements were performed using a VersaProbe II (Ulvac‐Phi, Chanhassen, USA). Al K_α_ and Mg K_α_ sources were used to investigate the powders.

Mössbauer spectra on selected powder samples were recorded in transmission geometry, using a ∼50 mCi ^57^Co radiation source mounted on a constant‐acceleration driving unit (WissEl GmbH, Ortenberg, Germany). Zero field spectra at low temperatures were recorded with a closed‐cycle cryostat (Lake Shore Cryotronics, Westerville, Ohio, USA) while a liquid helium bath cryostat with a superconducting split‐pair magnet was utilized for spectra recorded in an applied magnetic field of 5 T parallel to the γ‐ray propagation direction.

The macroscopic magnetic properties were characterized with the vibrating sample magnetometer (VSM) option of a PPMS DynaCool (Quantum Design Inc., San Diego, California, USA). Measurements on selected powder samples include magnetic field dependent M(H) loops up to ±9 T at 4.3 K and 300 K, as well as temperature dependent M(T) curves between 5 K and 380 K recorded in the standard zero field cooled – field cooled (ZFC‐FC) protocol at an applied magnetic field of 0.1 T.

High‐resolution scanning transmission electron microscopy (STEM) studies were carried out on a Jeol JEM 2200 fs microscope (Akishima, Japan) equipped with a probe‐side Cs‐corrector operated at 200 kV acceleration voltage. Micrographs were taken in conventional bright field as well as in high‐angle annular darkfield (HAADF) mode. In addition, EDX elemental mappings were acquired with an X‐Max 100 detector (Oxford Instruments, Abingdon, United Kingdom).

### 2‐Propanol adsorption and desorption DRIFTS

DRIFTS was performed using an FTIR spectrometer from ThermoFisher Scientific (Waltham, Massachusetts, USA), i. e., Nicolet™ iS20, equipped with a DRIFTS cell (Praying Mantis™, Harrick Scientific Products Inc., Pleasantville, New York, USA) and a Mercury Cadmium Telluride detector (MCT) cooled with liquid nitrogen. The DRIFTS cell outlet gas stream was analyzed continuously during the experiment by an online mass spectrometer (Omnistar GSD 320, Pfeiffer Vacuum, Wetzlar, Germany). Using a four‐port selector valve, the inlet gas was switched between two different streams, one for He‐purging and/or pretreatments (He or 20 % *v*/*v* O_2_ in He) and the other containing the probe gas (0.25 % *v*/*v* 2‐propanol in He). A home‐built saturator comprising of a double pipe heat exchanger and a submerged static mixer was used to provide 2‐propanol with the desired concentration. The temperature of the liquid 2‐propanol was maintained by external circulating chilled water flowing through the exchanger's outer tube, while the He flow was thoroughly dispersed in the liquid through the static mixer to maximize the contact between the gas and liquid. The gas streams were flowing through the catalyst bed inside the chamber in all segments with a total flowrate of 80 mLn min^−1^. At first, the loaded samples (∼30–40 mg fine powder) were pretreated by 20 % O_2_ in He at 350 °C for 1 h (at a heating rate of 10 °C min^−1^), after which the cell was cooled down to 20 °C at the maximum rate. After stabilizing the temperature, O_2_ was closed, the chamber and lines were purged with He for 10 min before collecting the background spectrum. Then the samples were exposed to 0.25 % 2‐propanol in He, and the spectra corresponding to the adsorption were collected for 40 min every 10 s. The first spectrum was collected before switching the gas, so it represents the time 0. Then the gas was automatically switched back to He to record the desorption spectra for another 40 min.

### Catalytic 2‐propanol oxidation

The measurements were performed in a home‐built apparatus consisting of a gas dosing unit (HovaGAS N G6 VOC−S, IAS GmbH, Germany) and a Tube Furnace MTF 10/25/130 (Carbolite Gero, Neuhausen, Germany), which is placed in a heating cabinet Thermocenter TC100 (SalvisLab, Rotkreuz, Switzerland). The reaction mixture, either going through or by‐passing the reactor, was analyzed on a downstream dual‐carrier gas Fusion Micro GC (Inficon GmbH, Bad Ragaz, Switzerland) equipped with four separation modules (2× Rt‐Molsieve 5 A, 1× Stabilwax DB, 1× Rt−Q Bond). 100 mg of the catalyst mixture was placed in a quartz reactor (inner diameter=8 mm).

The reaction consisted of three runs that were performed under the same temperature conditions. Only the reactive gas mixture was changed between the runs. Before each run, temperature‐programmed oxidation (TPO) up to 300 °C was performed at a flow rate of 50 mLn min^−1^ with an O_2_ concentration of 10 % (balanced in N_2_), the heating rate was set to 3 °C min^−1^. The maximum temperature was kept constant for 2 h. Afterward, the temperature was cooled down to 50 °C with a heating rate of 3 °C min^−1^. The temperature was kept constant for 65 min. During the first 20 min, the remaining O_2_ was purged out by N_2_ (100 mL min^−1^). In the last 45 min, the reaction mixture was purged for stabilization of the MFC flows. Afterward, the temperature was increased to 300 °C in the reaction mixture with a heating rate of 1 K min^−1^ up and held constant for 1 h. After the isothermal dwell, the temperature was cooled down to 50 °C with a heating rate of 1 K min^−1^ in the reaction mixture. Before the next TPO started, the temperature was dwelled for 10 min and a flow of 100 mL min^−1^ N_2_ was applied. The reaction mixture consisted of 0.169 % 2‐propanol and 0.169 % O_2_ balanced in N_2_ (dry mixture) for the first two runs and 0.175 % 2‐propanol, 0.172 % O_2,_ and 1.88 % H_2_O in N_2_ for the third run (wet mixture). The flow rate was kept constant at 100 mL min^−1^.

For steady‐state experiments, first, a TPO up to 300 °C was performed at a flow rate of 50 mLn min^−1^ with an O_2_ concentration of 10 % (balanced in N_2_), the heating rate was set to 3 °C min^−1^. The maximum temperature was kept constant for 2 h. Afterward, the temperature was cooled down to 50 °C with a heating rate of 3 °C min^−1^. The temperature was kept constant for 65 min. During the first 20 min, the remaining O_2_ was purged out by N_2_ (100 mL min^−1^). In the last 45 min, the reaction mixture was purged for stabilization of the MFC flows. Afterward, the temperature was increased to 290 °C (for x=0.00) or 190 °C (for x=0.25) in the dry mixture with a heating rate of 1 K min^−1^ up and held constant for 24 h. After the isothermal dwell, the temperature was cooled down to 50 °C with a heating rate of 1 K min^−1^ in the dry reaction mixture. Afterward, another cycle with the same temperature and flow conditions was performed. The only difference was the use of the wet instead of the dry reaction mixture.

### Near ambient pressure XPS studies

Near ambient pressure X‐ray photoelectron spectroscopy (NAP‐XPS) was performed at the UE56/2‐PGM1 (Elliptical Undulator) beamline of the synchrotron radiation facility BESSY II of Helmholtz‐Zentrum Berlin, Germany. The used end‐station consisted of a near‐ambient pressure (NAP) photoelectron analyzer provided by SPECS GmbH (Phoibos 150). To minimize losses of photons and electrons a 50 nm thick SiN_x_ X‐ray membrane close to the sample and a differentially pumped electron analyzer with electrostatic lenses were used. Also, it is equipped with a sapphire sample holder, where typically a powder pellet and K‐type thermocouple are fixed. The sapphire sample holder is mounted inside the XPS setup near to the aperture of the first differential pumping stage. The heating treatment is carried out through an infrared laser from the rear. The gas composition during the *operando* measurements was monitored by a quadrupole mass spectrometer. Details of the experimental setup can be found in the literature.^[58]^

The pellet was pretreated by annealing in 0.25 mbar O_2_ at 250 °C using a heating rate of 5 °C min^−1^ to clean the sample from adsorbed carbon species. Afterward, cooling of the sample to 100 °C was performed and for the dry feed a mixture of 2‐propanol, oxygen and argon was introduced (0.25 mbar, 1 mLn min^−1^ : 1 mLn min^−1^ : 0.15 mLn min^−1^) into the XPS cell. Each partial pressure is controlled by mass flow controllers. Then, the temperature was increased with a heating rate of 10 °C min^−1^ up to 200 °C at which the XPS measurements were performed. All core level regions were recorded with a kinetic energy of 150 eV.

For the measurements in wet feed a fresh pellet was used. The procedure was the same as described above, but the reaction gas mixture consisted of water, 2‐propanol, oxygen, and argon (0.25 mbar, 0.7 mLn min^−1^ : 0.7 mLn min^−1^ : 0.7 mLn min^−1^ : 0.1 mLn min^−1^).

XPS spectra were analyzed through CasaXPS and Igor Pro. The binding energies were calibrated to the fermi edge of a Pd reference sample. The O 1s and C 1s spectra were deconvoluted with combined Gaussian and Lorentzian functions after a Shirley+linear background subtraction.

## Conflict of interest

The authors declare no conflict of interest.

## Supporting information

As a service to our authors and readers, this journal provides supporting information supplied by the authors. Such materials are peer reviewed and may be re‐organized for online delivery, but are not copy‐edited or typeset. Technical support issues arising from supporting information (other than missing files) should be addressed to the authors.

Supporting Information
