# Supplementary material for: The Effect of Water on the 2‐Propanol Oxidation Activity of Co‐Substituted LaFe1−xCoxO3 Perovskites
Source: Chemistry. 2021 Nov 8;27(68):17127–44. doi: 10.1002/chem.202102791 (PMC9299464; doi:10.1002/chem.202102791)
Supplement: Supplementary file 1 — Supporting Information [file CHEM-27-17127-s001.pdf]

Chemistry–A European Journal

Supporting Information

The Effect of Water on the 2-Propanol Oxidation Activity of Co-Substituted $LaFe_{1-}Co_xO_3$ Perovskites

Chemistry–A European Journal

Supporting Information

The Effect of Water on the 2-Propanol Oxidation Activity of Co-Substituted $LaFe_{1-}Co_xO_3$ Perovskites

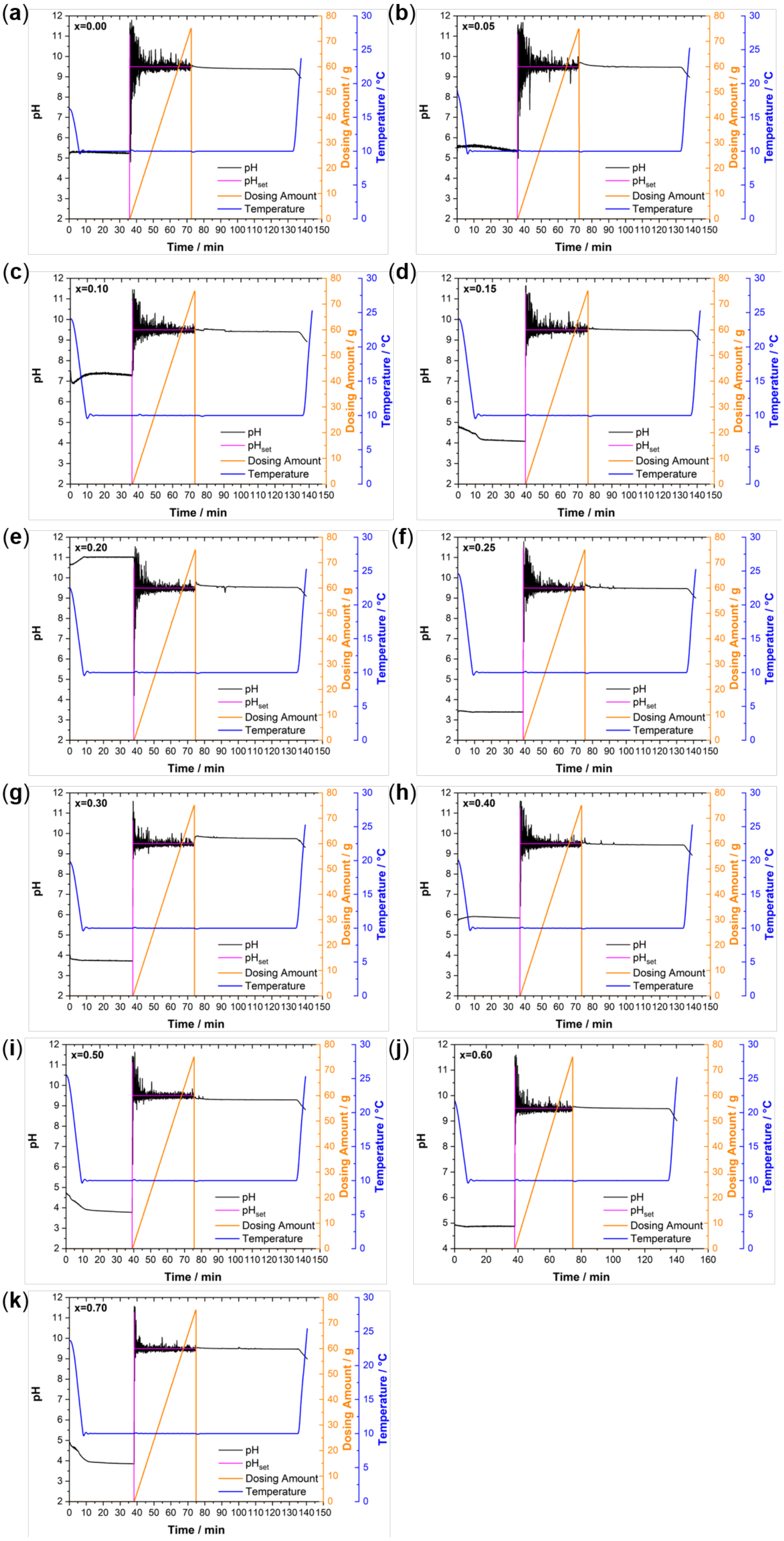

Figure S1: Synthesis protocols of (a) x=0.00, (b) x=0.05, (c) x=0.10, (d) x=0.15, (e) x=0.20, (f) x=0.25, (g) x=0.30, (h) x=0.40, (i) x=0.50, (j) x=0.60 and (k) x=0.70.

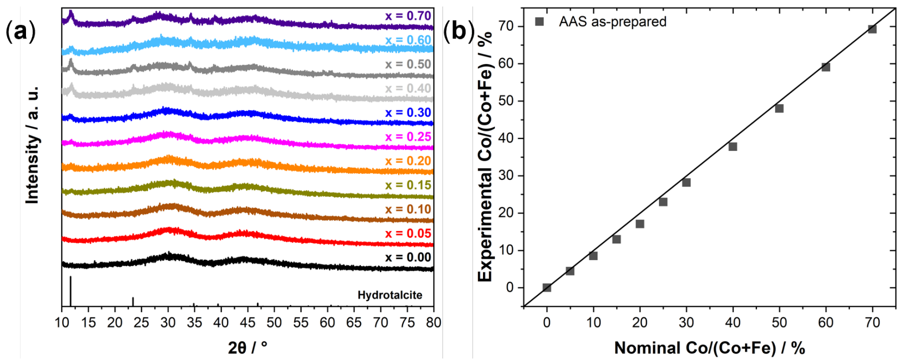

Figure S2: (a) XRD patterns of the co-precipitated precursor materials. (b) Experimental ratio Co/(Co+Fe) derived from AAS of the precursors.

Table S1. Criteria of fit for the Rietveld refinements of the X-ray diffraction patterns of the calcined samples. In case of the R_{Bragg} vales, the indicator O is the abbreviation for the orthorhombic perovskite, R for the rhombohedral perovskite and S for the spinel phase.

x=	R_{exp}	R_{wp}	Rp	R_{exp}'	R_{wp}'	R_p'	GOF	$R_{Bragg,O}$	$R_{Bragg,R}$	$R_{Bragg,S}$
0.00	10.98	12.54	8.61	5.65	6.45	5.09	1.14	2.051	–	–
0.05	9.55	12.16	8.1	5	6.37	5.09	1.27	2.966	–	–
0.10	10.55	13.09	8.62	5.19	6.44	5.08	1.24	3.368	–	–
0.15	9.2	13.01	8.89	4.75	6.72	5.5	1.41	4.1	–	–
0.20	9.17	13.33	9.17	4.81	7	5.7	1.45	4.625	–	–
0.25	9.76	14.68	10.05	4.87	7.33	5.99	1.5	5.301	–	–
0.30	8.66	14.99	10.27	4.75	8.23	6.57	1.73	5.898	–	–
0.40	10.71	15.11	10.73	5.03	7.1	6.08	1.41	4.202	4.084	5.196
0.50	10.05	15.75	11.46	4.91	7.69	6.68	1.57	4.34	5.829	4.202
0.60	9.52	14.54	10.64	5.03	7.68	6.59	1.53	2.703	3.172	4.429
0.70	9.98	13.75	10.31	5.62	7.74	6.61	1.38	3.531	3.362	3.229

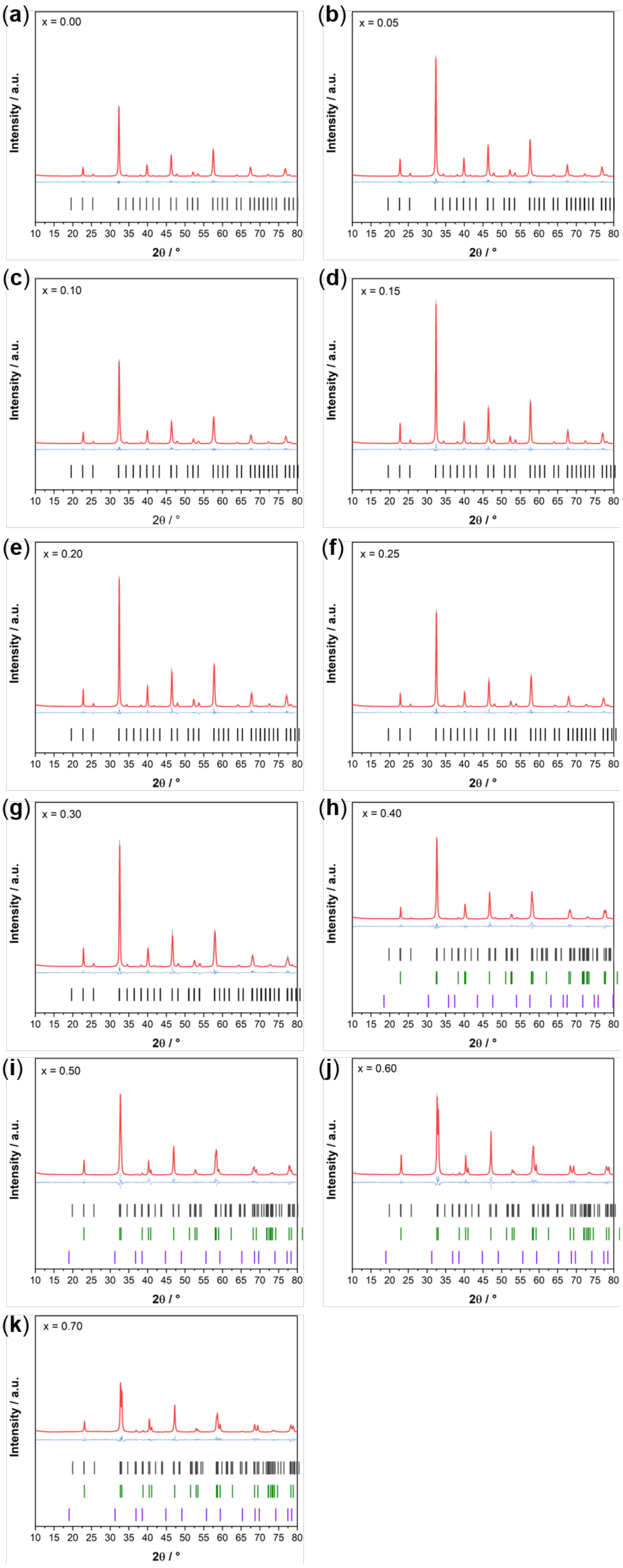

Figure S3: Rietveld refinement plots of (a) x=0.00, (b) x=0.05, (c) x=0.10, (d) x=0.15, (e) x=0.20, (f) x=0.25, (g) x=0.30, (h) x=0.40, (i) x=0.50, (j) x=0.60 and (k) x=0.70.

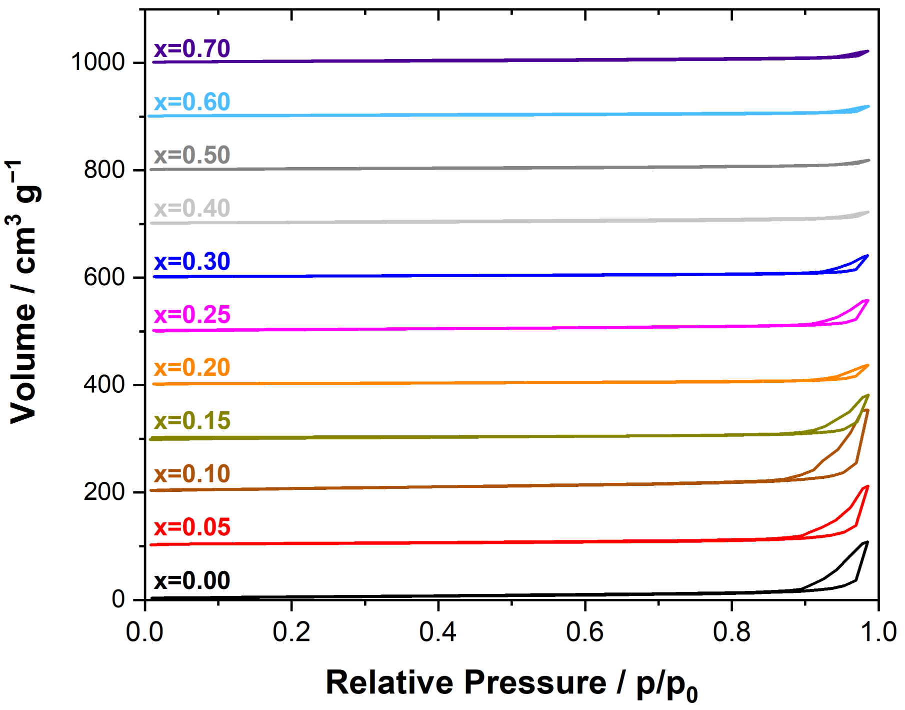

Figure S4: N_2 adsorption isotherms.

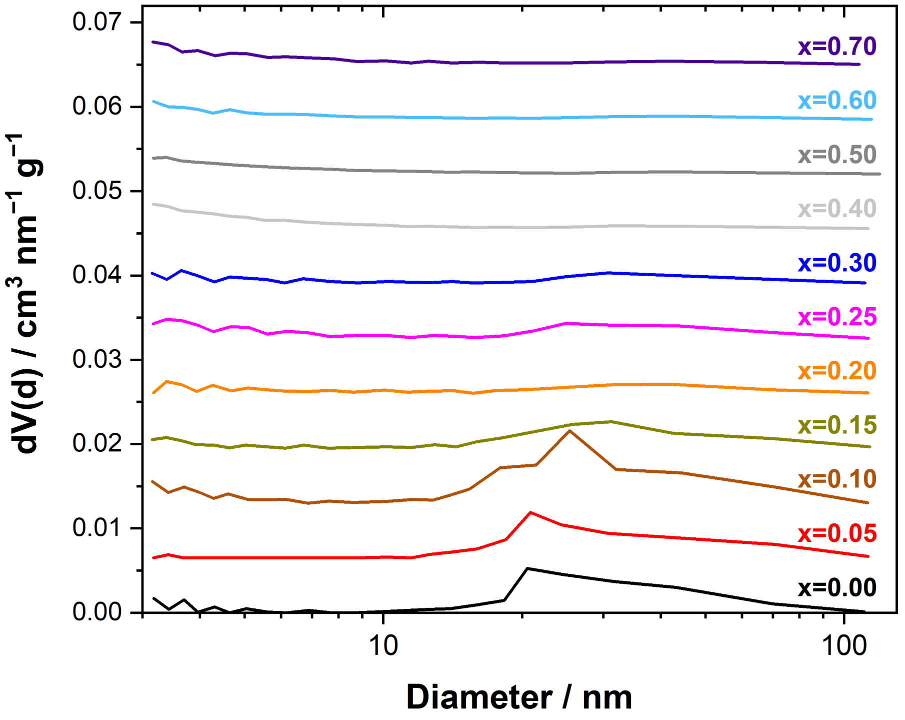

Figure S5: Pore size distributions determined by the BJH method during desorption.

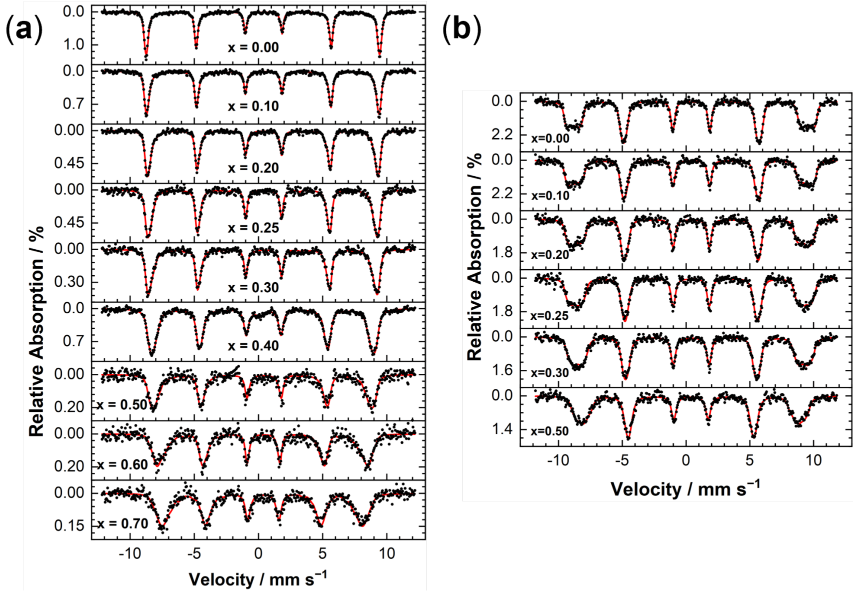

Figure S6: Mössbauer spectra (black dots) and fits (red lines) for 5 K zero field (a) and 4.3 K 5 T (b) measurements. Zero field spectra were reproduced via narrow distributions of the hyperfine field B_{HF}, while in-field spectra were fitted using distributions of the canting angle between the applied magnetic field and spin direction. Spectral structure and line intensities are indicative of a canted antiferromagnetic (weakly FM) structure.

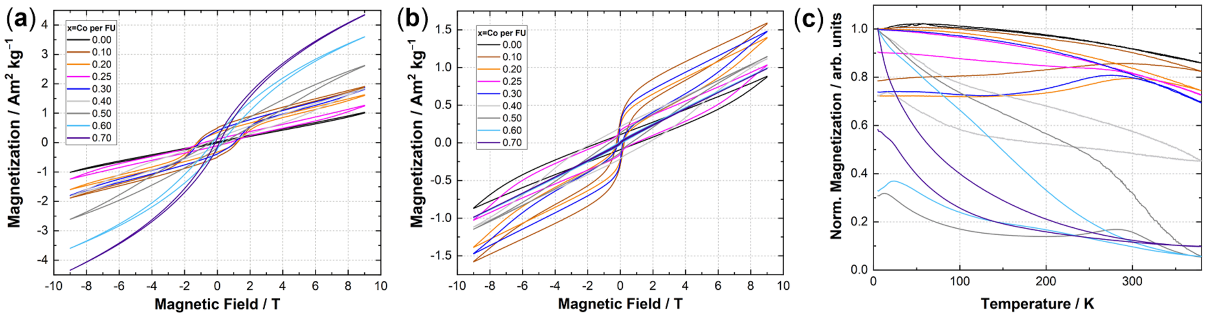

Figure S7: (a) 9 T M(H) magnetization curves recorded at 4.3 K. (b) 9 T M(H) magnetization curves recorded at 300 K. (c) Temperature dependent magnetization curves recorded between 5 K and 380 K at 0.1 T using the standard zero field cooled – field cooled (ZFC-FC) protocol.

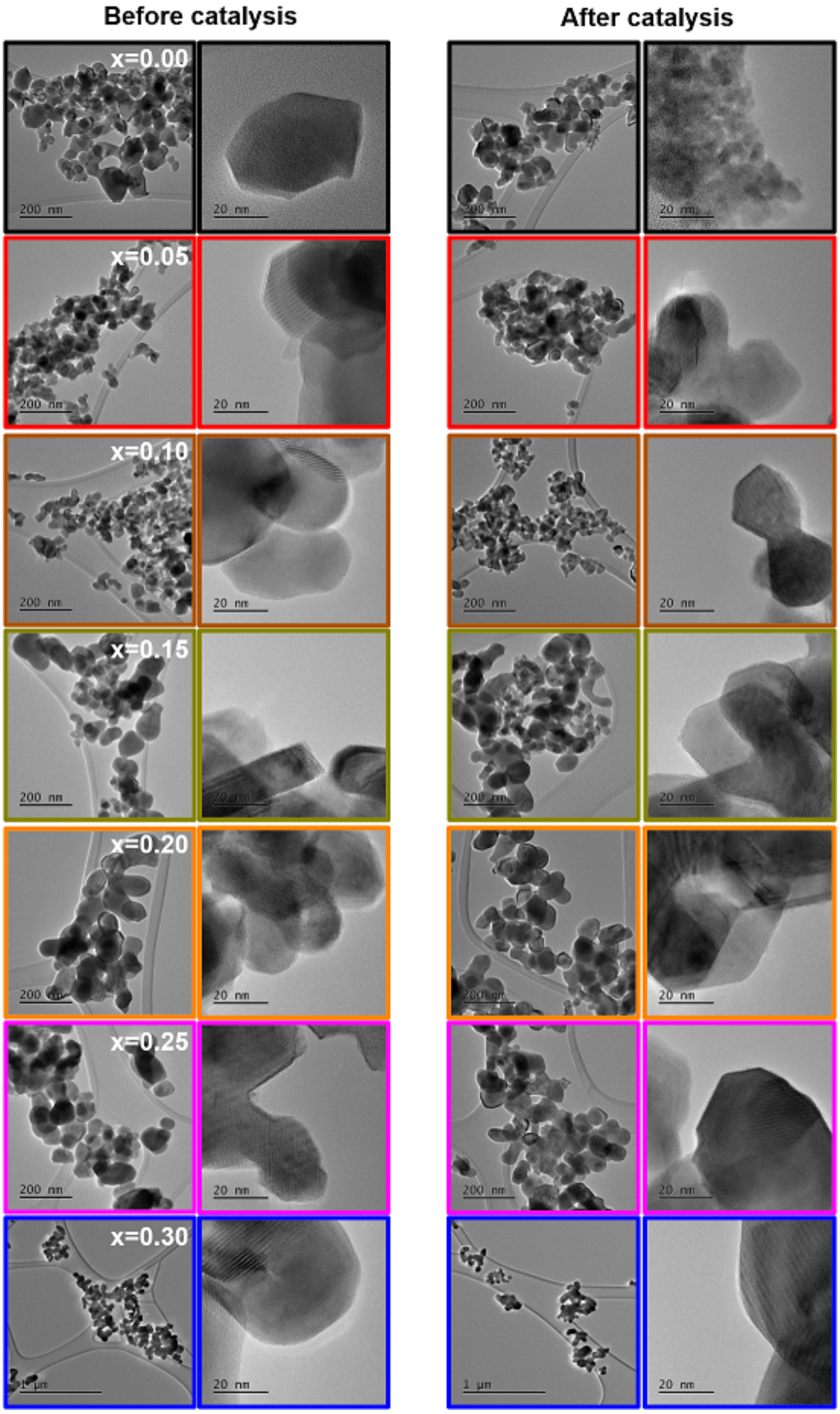

Figure S8: TEM micrographs before and after catalysis for $0.00 \leq x \leq 0.30$.

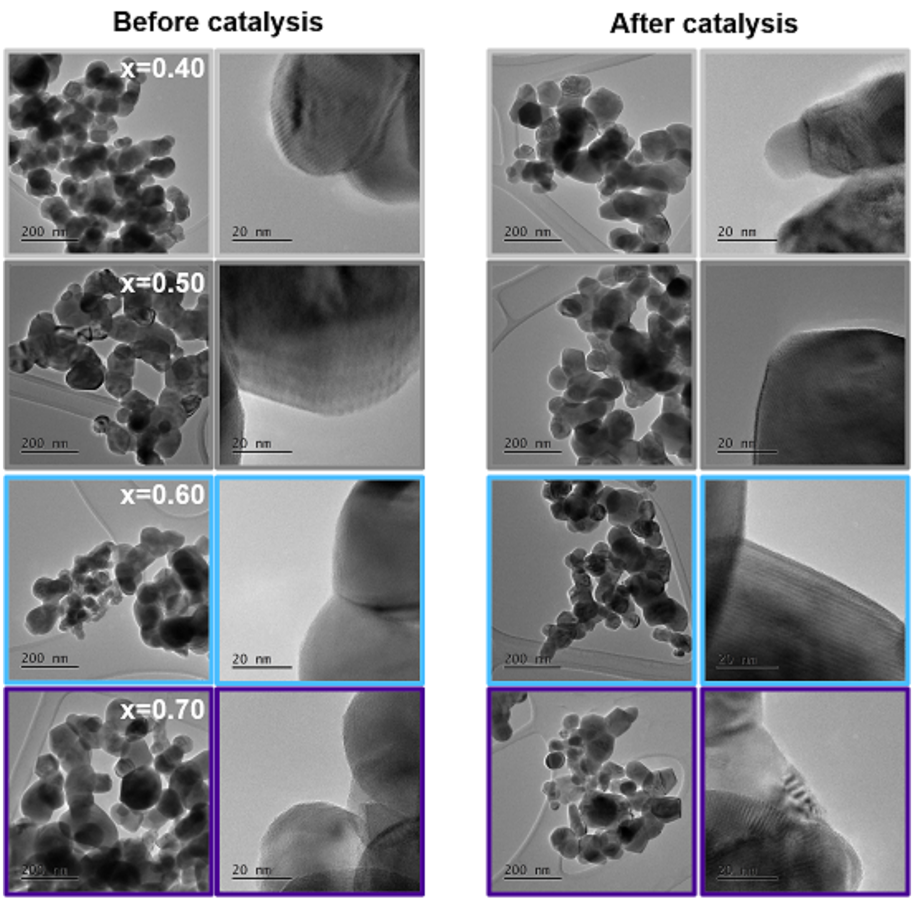

Figure S9: TEM micrographs before and after catalysis for $0.40 \leq x \leq 0.70$.

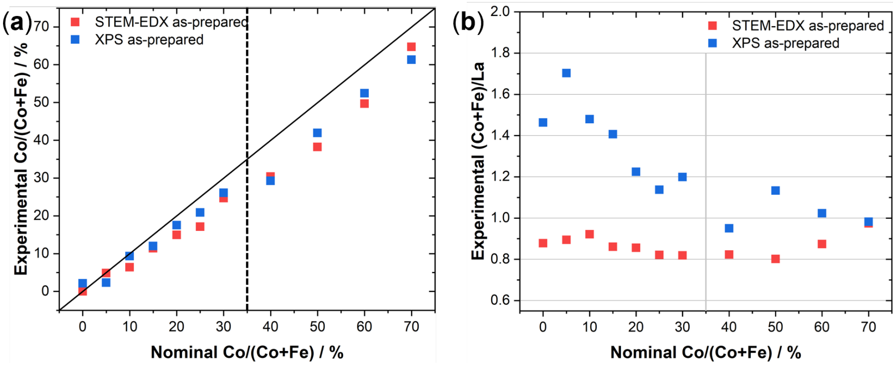

Figure S10: (a) Experimental ratio Co/(Co+Fe) derived from STEM-EDX and XPS. (b) Experimental ratio (Co+Fe)/La derived from STEM-EDX and XPS. The term as-prepared refers to the calcined material prior to any catalytic experiment.

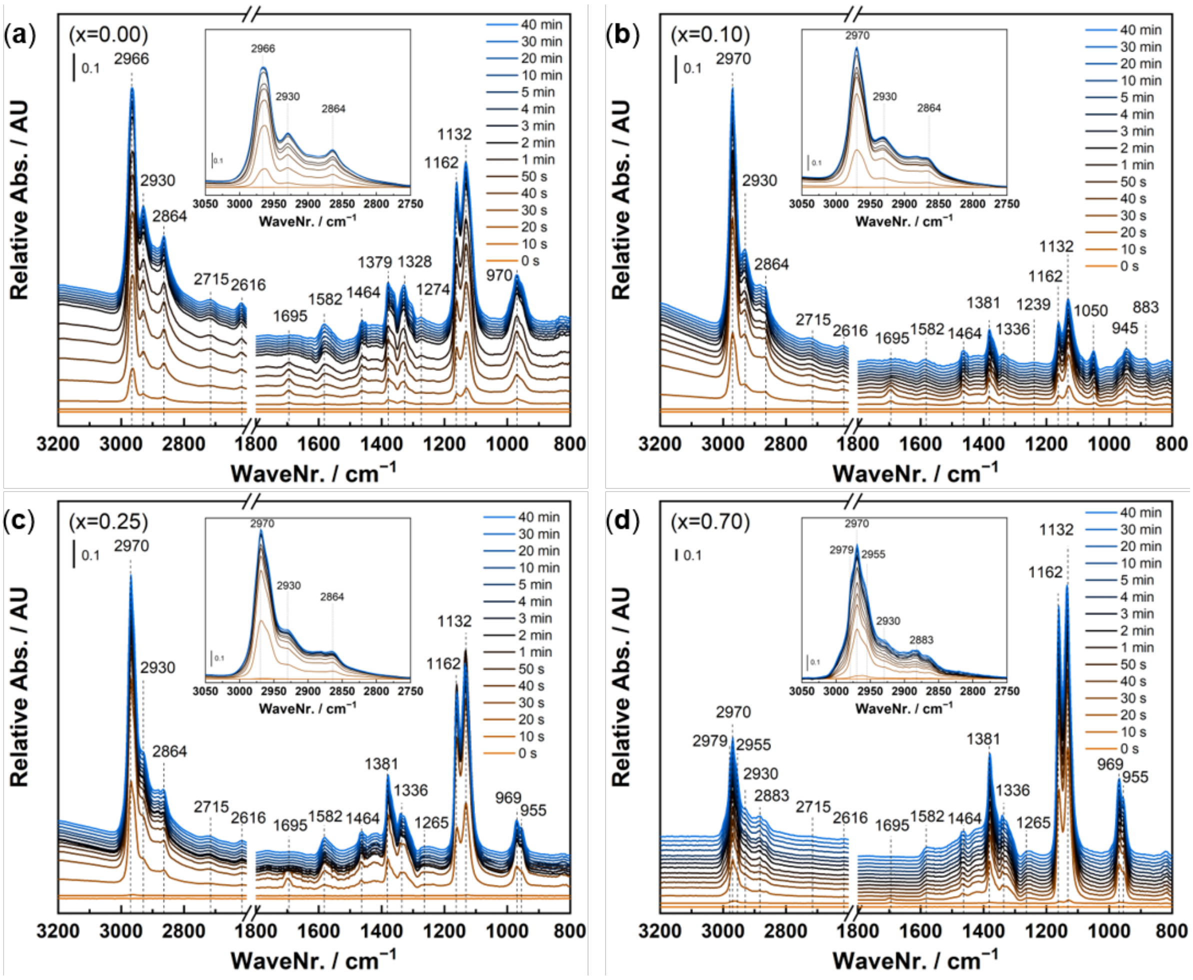

Figure S11: Stacked time-dependent DRIFTS spectra during 2-propanol adsorption for (a) x=0.00, (b) x=0.10, (c) x=0.25, and (d) x=0.70. The insets show the non-stacked spectra for the wavenumber range corresponding to the CH_3 and CH stretching vibrations.

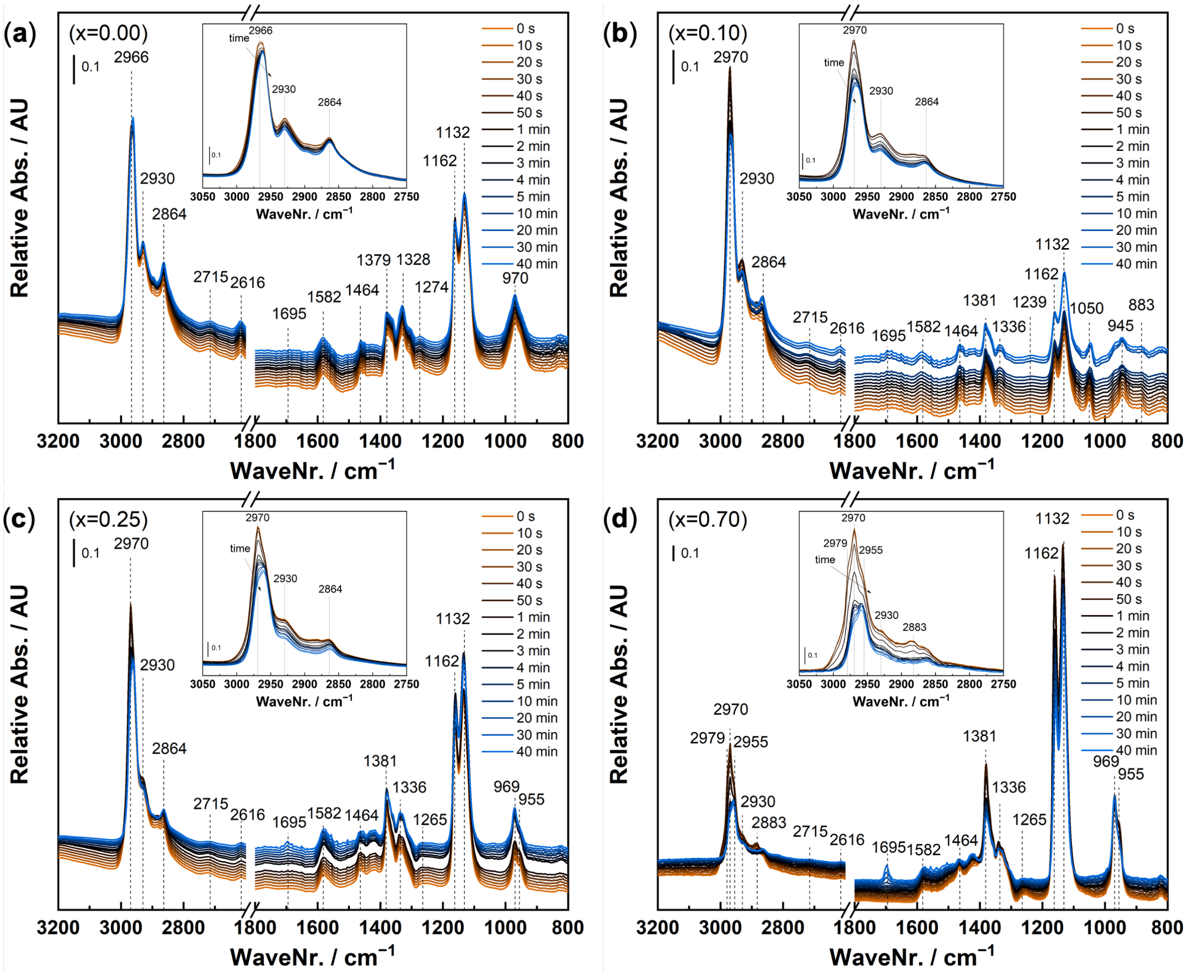

Figure S12: Stacked time-dependent DRIFTS spectra during 2-propanol desorption for (a) x=0.00, (b) x=0.10, (c) x=0.25, and (d) x=0.70. The insets show the non-stacked spectra for the wavenumber range corresponding to the CH_3 and CH stretching vibrations

Table S2: Summary of the FTIR bands and their assignments (ν: stretching, δ: scissoring, ρ: rocking modes of vibrations with "as" and "s" denoting the asymmetric and symmetric respectively).

Band position / cm^{-1}	Assignment
2967	$\nu\ (C-H_3)as$
2931	$\nu\ (C-H_3)s$
2865	$\nu\ (C-H)$
1693	$\nu\ (C=O)$
1590	$\nu\ (C=C)$
1464	$\delta\ (C-H_3)as$
1381	$\delta\ (C-H_3)s$
1333	$\delta\ (C-H)$
1242	$\delta\ (O-H)$
1163	$\nu\ (C-C)$
1130	$\rho\ (C-H_3)$ and $\nu(C-O)$

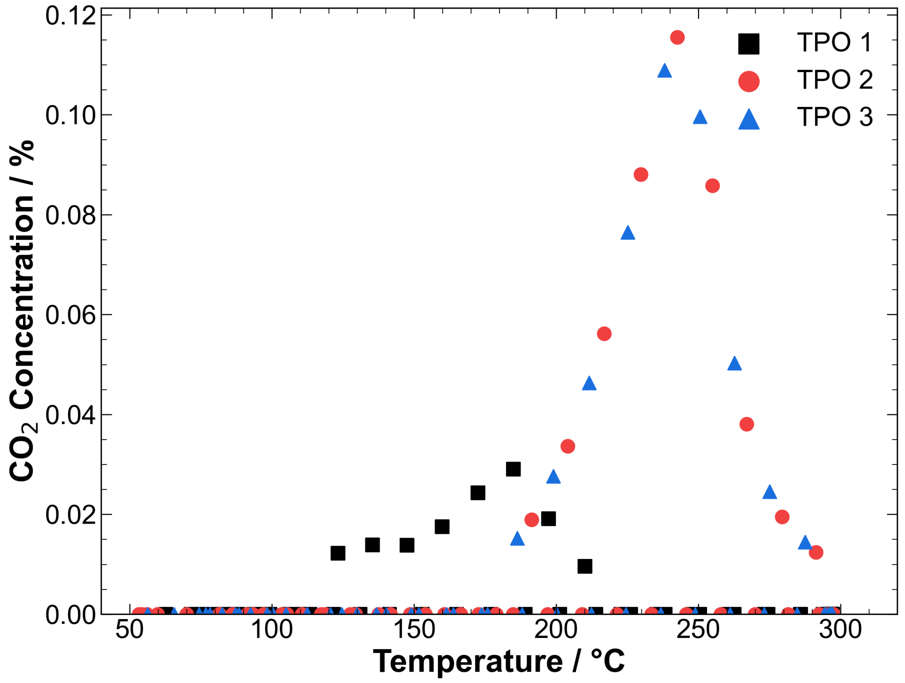

Figure S13: CO_2 concentrations during TPO experiments for x=0.25.

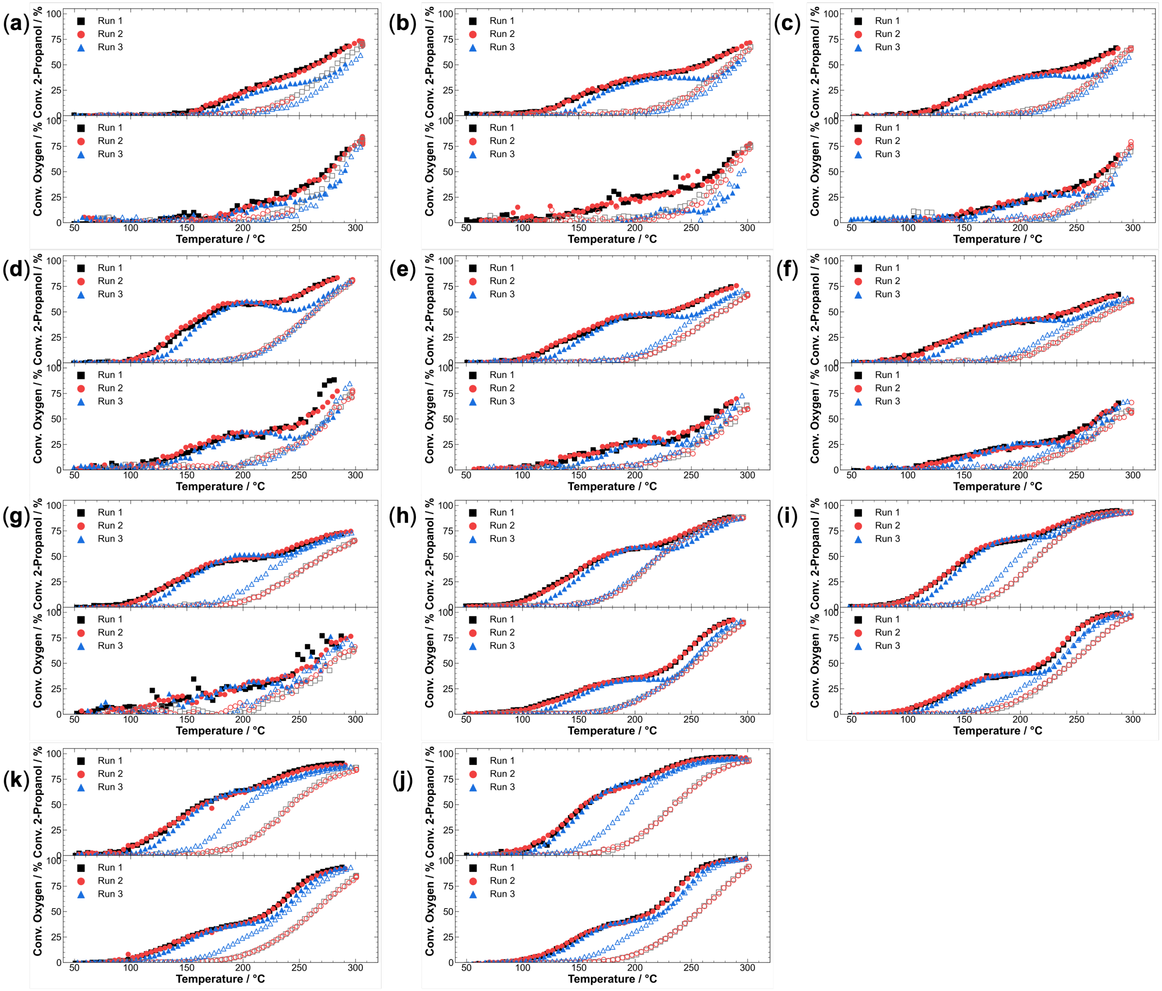

Figure S14: 2-Propanol and oxygen conversion during the dry (Run 1 and Run 2) and wet feed (Run 3) runs for (a) x=0.00, (b) x=0.05, (c) x=0.10, (d) x=0.15, (e) x=0.20, (f) x=0.25, (g) x=0.30, (h) x=0.40, (i) x=0.50, (j) x=0.60 and (k) x=0.70. Filled symbols show data points during heating, empty characters show the behavior during cooling.

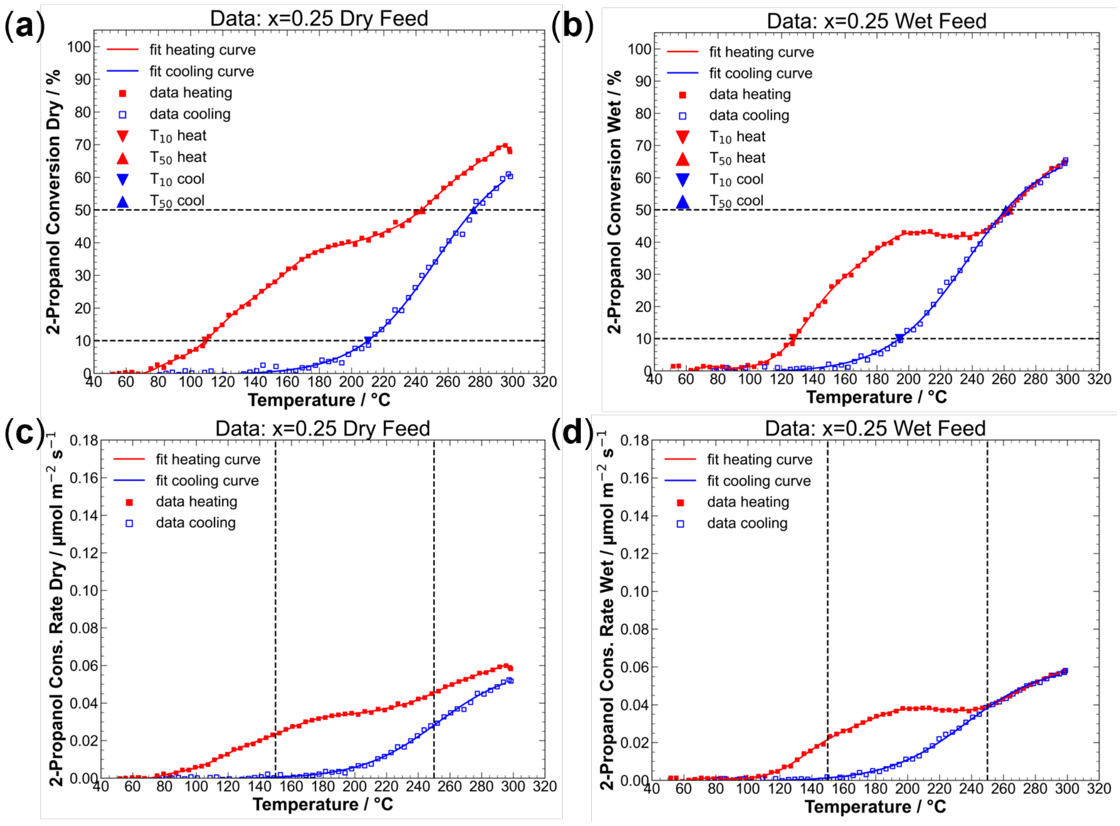

Figure S15: 2-Propanol conversions during heating and cooling and corresponding fits for x=0.25 in (a) dry feed and (b) wet feed. 2-Propanol consumption rate fits for x=0.25 in (c) dry feed and (d) wet feed.

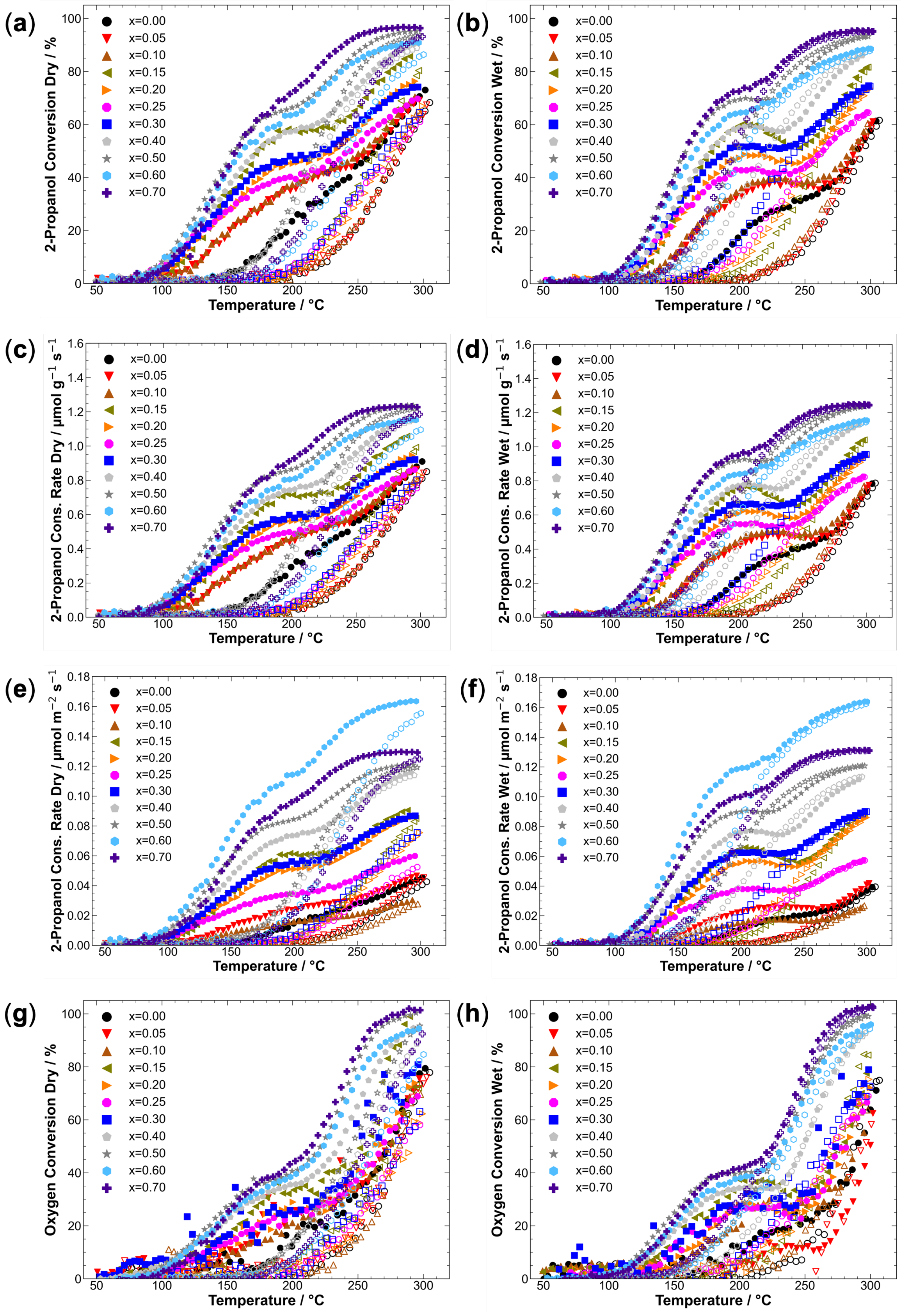

Figure S16: (a) 2-Propanol conversions in dry feed. (b) 2-Propanol conversions in wet feed. (c) Weight normalized 2-propanol consumption rates in dry feed. (d) Weight normalized 2-propanol consumption rates in wet feed. (e) Surface area normalized 2-propanol consumption rates in dry feed. (f) Surface area normalized 2-propanol consumption rates in wet feed. (g) Oxygen conversions in dry feed. (h) Oxygen conversions in wet feed. Filled symbols show data points during heating, empty characters show the behavior during cooling.

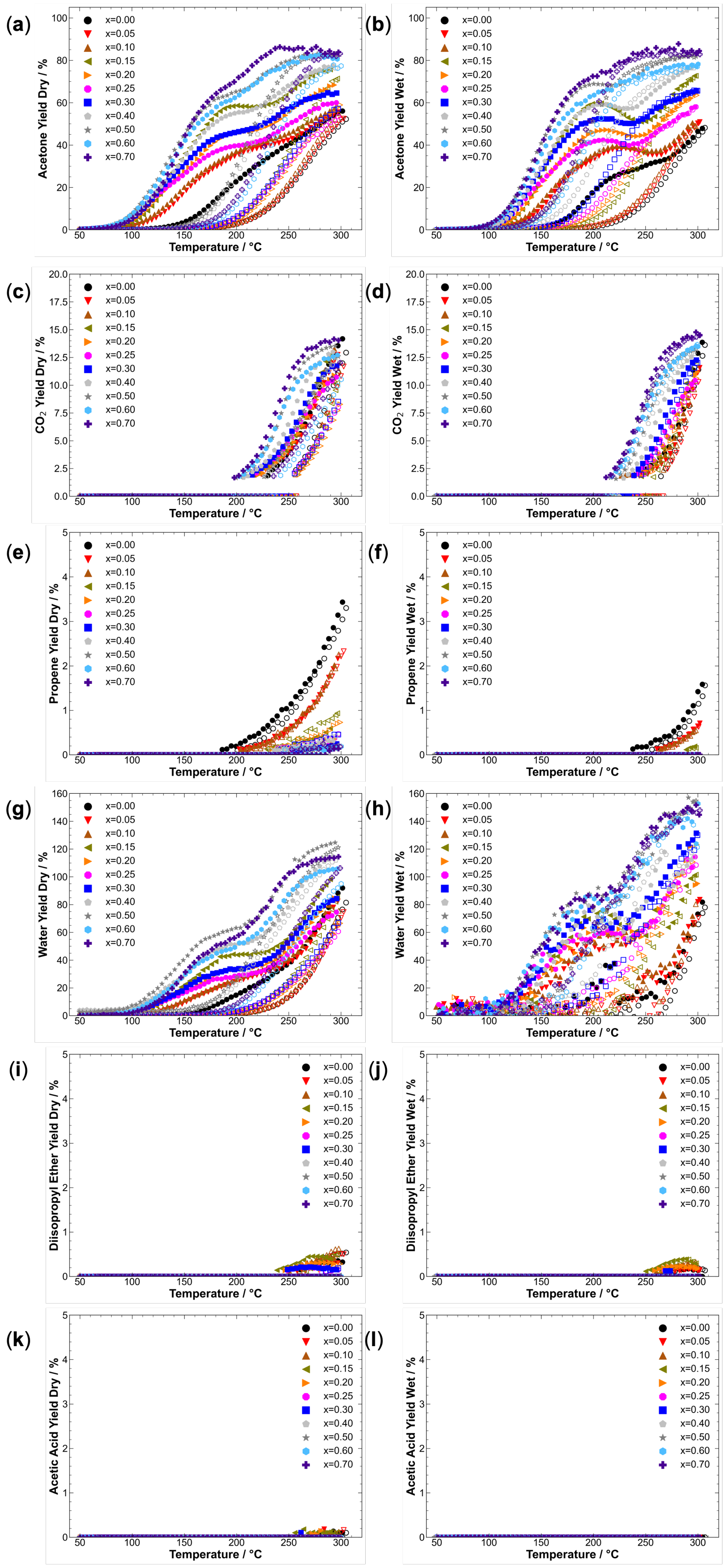

Figure S17: (a) Acetone yields in dry feed. (b) Acetone yields in wet feed. (c) CO_2 yields in dry feed. (d) CO_2 yields in wet feed. (e) Propene yields in dry feed. (f) Propene yields in wet feed. (g) Water yields in dry feed. (h) Water yields in wet feed. (i) Diisopropylether yields in dry feed. (j) Diisopropylether yields in wet feed. (k) Acetic acid yields in dry feed. (l) Acetic acid yields in wet feed. Filled symbols show data points during heating, empty characters show the behavior during cooling.

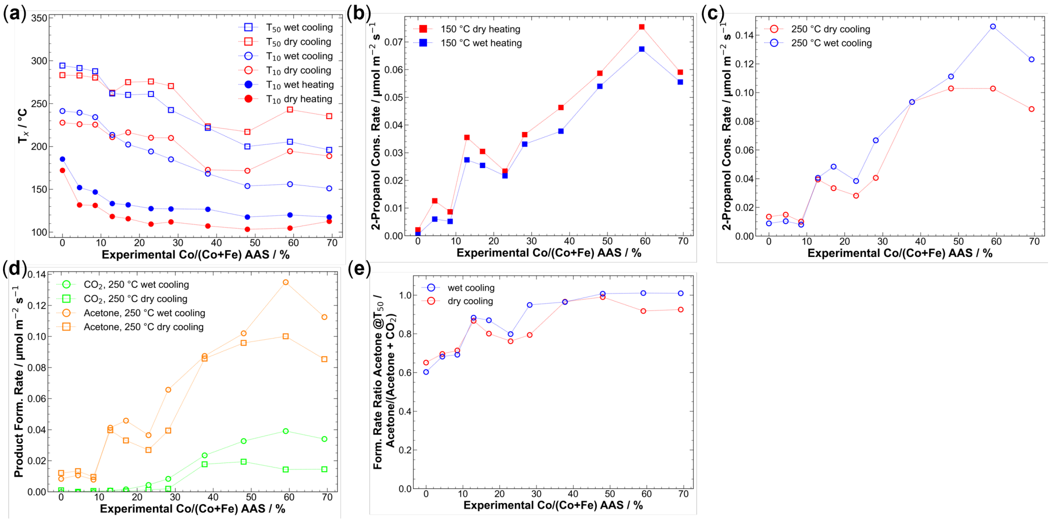

Figure S18: (a) T_{10} values during heating and cooling and T_{50} during cooling in dry and wet feed plotted against the experimental Co content derived from AAS. (b) Surface-area normalized 2-propanol consumption rates at 150 °C during dry and wet feed heating plotted against the experimental Co content derived from AAS. (c) Surface-area normalized 2-propanol consumption rates at 250 °C during dry and wet feed cooling plotted against the experimental Co content derived from AAS.

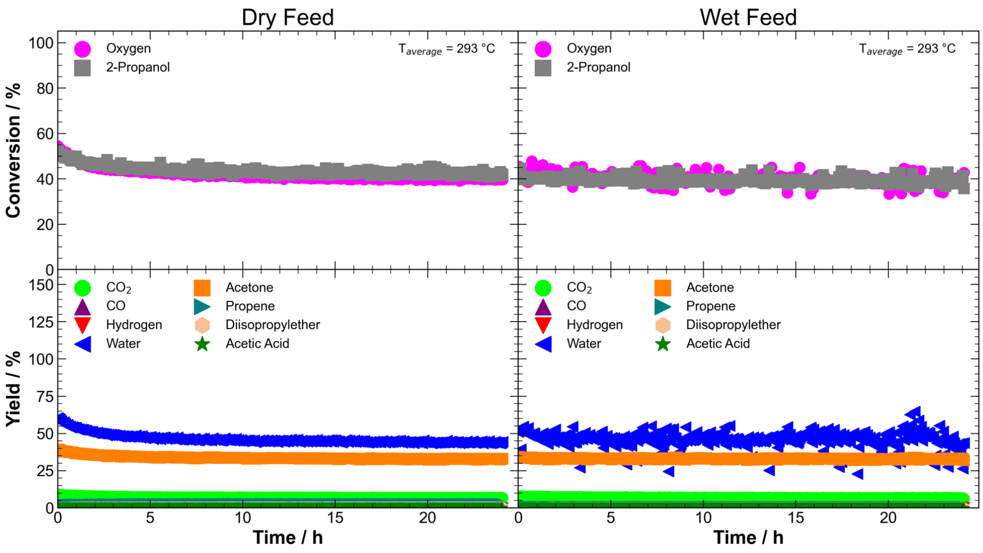

Figure S19: Stability dataset for x=0.00; conversions of 2-propanol and oxygen (top row) and product yields (bottom row) during dry and wet runs.

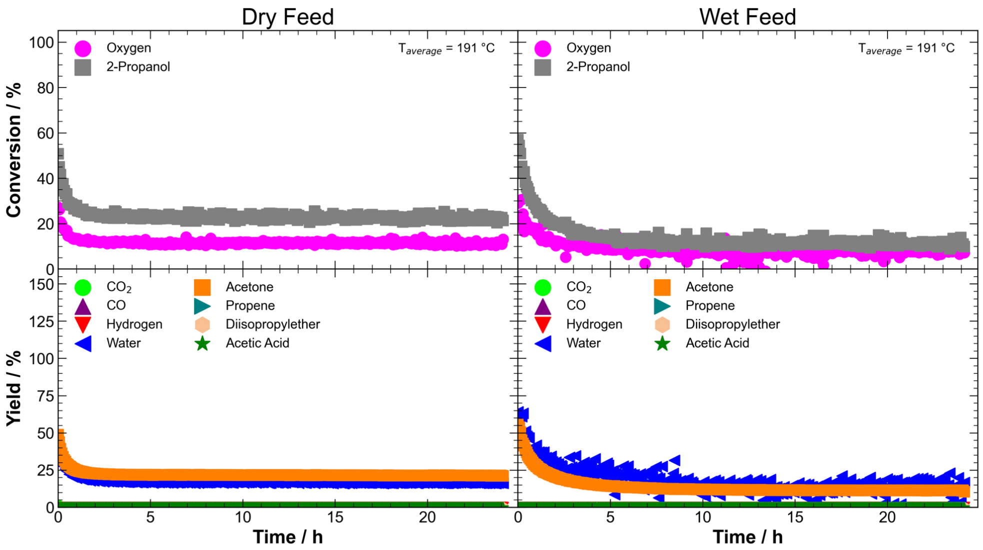

Figure S20: Stability dataset for x=0.25; conversions of 2-propanol and oxygen (top row) and product yields (bottom row) during dry and wet runs.

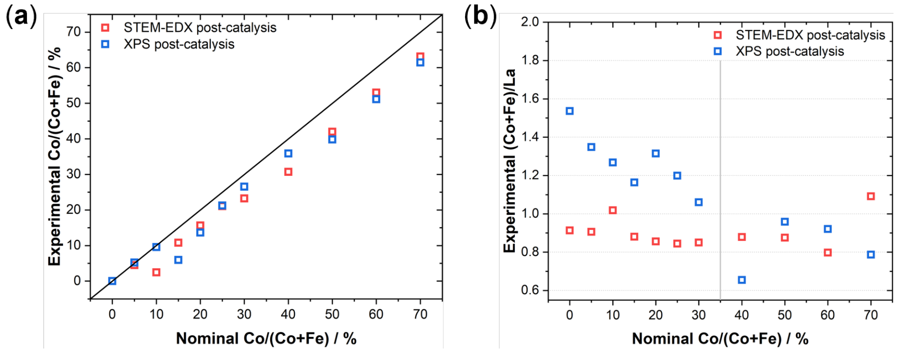

Figure S21: (a) Comparison of nominal and experimental Co content derived from (S)TEM-EDX and XPS after catalysis. (b) Ratio of B-cations to A-cations derived from (S)TEM-EDX and XPS after catalysis.

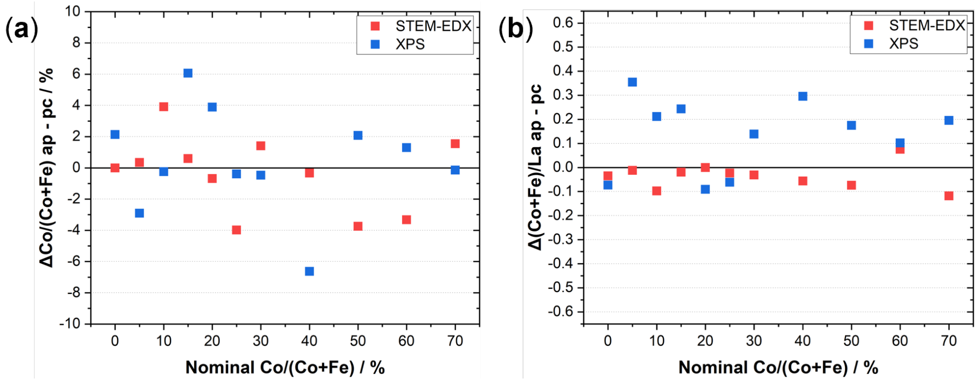

Figure S22: (a) Differences in experimental Co content between the cases of as-prepared (ap) and post-catalysis (pc). (b) Differences in ratio of B-cations to A-cations between as-prepared (ap) and post-catalysis (pc).

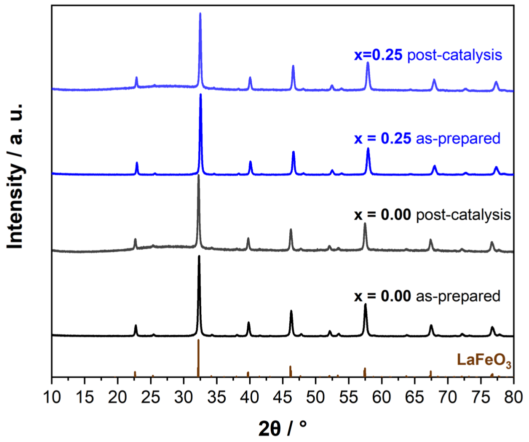

Figure S23: XRD patterns of x=0.00 and x=0.25 in the as-prepared state and in the post-catalysis state after the steady-state measurements shown in Figure S19 and Figure S20.

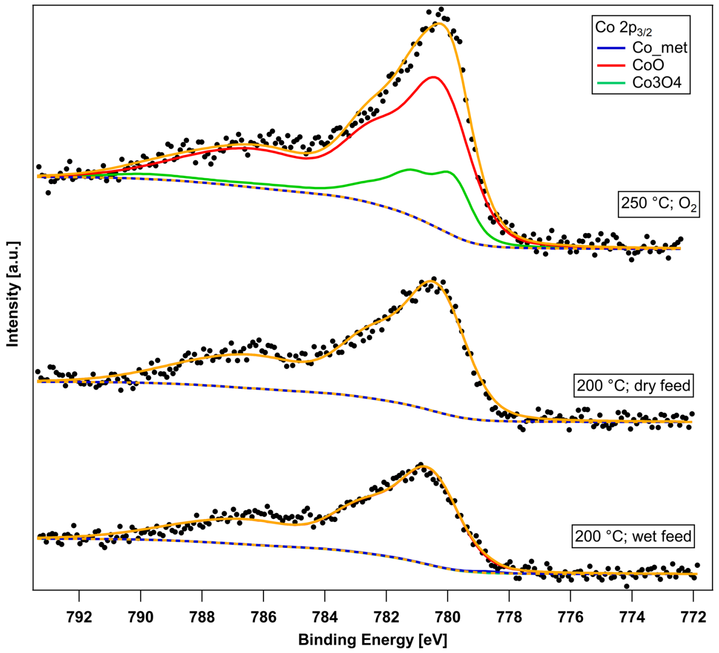

Figure S24: Linear combination fits (see main text) to determine the occupancy of different Co oxidation states during dry feed and wet feed as well as during the pre-treatment step at 250 °C in 0.25 mbar O_2. For the wet and dry feed, the Co is purely CoO, seen by an overlap of the red line of the CoO components with the overall fitting curve (orange).
